# Heterogeneous recurrent spiking neural network for spatio-temporal classification

**DOI:** 10.3389/fnins.2023.994517

**Published:** 2023-01-30

**Authors:** Biswadeep Chakraborty, Saibal Mukhopadhyay

**Affiliations:** Department of Electrical and Computer Engineering, Georgia Institute of Technology, Atlanta, GA, United States

**Keywords:** spiking neural network (SNN), action detection and recognition, spike timing dependent plasticity, heterogeneity, unsupervised learning, Bayesian Optimization (BO), leaky integrate and fire (LIF)

## Abstract

Spiking Neural Networks are often touted as brain-inspired learning models for the third wave of Artificial Intelligence. Although recent SNNs trained with supervised backpropagation show classification accuracy comparable to deep networks, the performance of unsupervised learning-based SNNs remains much lower. This paper presents a heterogeneous recurrent spiking neural network (HRSNN) with unsupervised learning for spatio-temporal classification of video activity recognition tasks on RGB (KTH, UCF11, UCF101) and event-based datasets (DVS128 Gesture). We observed an accuracy of 94.32% for the KTH dataset, 79.58% and 77.53% for the UCF11 and UCF101 datasets, respectively, and an accuracy of 96.54% on the event-based DVS Gesture dataset using the novel unsupervised HRSNN model. The key novelty of the HRSNN is that the recurrent layer in HRSNN consists of heterogeneous neurons with varying firing/relaxation dynamics, and they are trained via heterogeneous spike-time-dependent-plasticity (STDP) with varying learning dynamics for each synapse. We show that this novel combination of heterogeneity in architecture and learning method outperforms current homogeneous spiking neural networks. We further show that HRSNN can achieve similar performance to state-of-the-art backpropagation trained supervised SNN, but with less computation (fewer neurons and sparse connection) and less training data.

## 1. Introduction

Acclaimed as the third generation of neural networks, spiking neural networks (SNNs) have become very popular. In general, SNN promises lower operating power when mapped to hardware. In addition, recent developments of SNNs with leaky integrate-and-fire (LIF) neurons have shown classification performance similar to deep neural networks (DNN). However, most of these works use supervised statistical training algorithms such as backpropagation-through-time (BPTT) (Jin et al., 2018; Shrestha and Orchard, 2018; Wu et al., 2018). These backpropagated models are extremely data-dependent and show poor trainability with less training data, and generalization characteristics (Tavanaei et al., 2019; Lobo et al., 2020). In addition, BPTT-trained models need highly complex architecture with a large number of neurons for good performance. Though unsupervised learning methods like the STDP have been introduced, they lack performance compared to their backpropagated counterparts. This is attributed to the high training complexity of these STDP dynamics (Lazar et al., 2006). Therefore, there is a need to explore SNN architectures and algorithms that can improve the performance of unsupervised learned SNN.

This paper introduces a Heterogeneous Recurrent Spiking Neural Network (HRSNN) with heterogeneity in both the LIF neuron parameters and the STDP dynamics between the neurons. Recent works have discussed that heterogeneity in neuron time constants improves the model's performance in the classification task (Perez-Nieves et al., 2021; She et al., 2021b; Yin et al., 2021; Zeldenrust et al., 2021). However, these papers lack a theoretical understanding of why heterogeneity improves the classification properties of the network. Current literature primarily looks into how heterogeneity in neuronal timescales improves the model performance. They do not study how heterogeneity can be leveraged to engineer sparse neural networks. In addition, the previous papers do not study the effect of heterogeneity on the amount of training data needed for the model. In this paper, we studied how the heterogeneity in both the neuronal and synaptic parameters can help us engineer models that can perform well with less training data and fewer synaptic connections.

Our work also uses a novel BO method to optimize the hyperparameter search process, making it highly scalable for larger heterogeneous networks that can be used for more complex tasks like action recognition, which was not possible earlier. First, we analytically show that heterogeneity improves the linear separation property of unsupervised SNN models. We also empirically verified that heterogeneity in the LIF parameters and the STDP dynamics significantly improves the classification performance using fewer neurons, sparse connections, and lesser training data. We use a Bayesian Optimization (BO)-based method using a modified Matern Kernel on the Wasserstein metric space to search for optimal parameters of the HRSNN model and evaluate the performance on RGB (KTH, UCF11, and UCF101) and event-based datasets (DVS-Gesture). The HRSNN model achieves an accuracy of 94.32% on KTH, 79.58% on UCF11, 77.33% on UCF101, and 96.54% on DVS-Gesture using 2,000 LIF neurons.

## 2. Related works

### 2.1. Recurrent spiking neural network

#### 2.1.1. Supervised learning

Recurrent networks of spiking neurons can be effectively trained to achieve competitive performance compared to standard recurrent neural networks. Demin and Nekhaev (2018) showed that using recurrence could reduce the number of layers in SNN models and potentially form the various functional network structures. Zhang and Li (2019) proposed a spike-train level recurrent SNN backpropagation method to train the deep RSNNs, which achieves excellent performance in image and speech classification tasks. On the other hand, Wang et al. (2021) used the recurrent LIF neuron model with the dynamic presynaptic currents and trained by the BP based on surrogate gradient. Some recent works introduces heterogeneity in the LIF parameters using trainable time constants (Fang et al., 2021). However, these methods are supervised learning models and also do not scale with a greater number of hyperparameters.

#### 2.1.2. Unsupervised learning

Unsupervised learning models like STDP have shown great generalization, and trainability properties (Chakraborty and Mukhopadhyay, 2021). Previous works have used STDP for training the recurrent spiking networks (Gilson et al., 2010). Nobukawa et al. (2019) used a hybrid STDP and Dopamine-modulated STDP to train the recurrent spiking network and showed its performance in classifying patterns. Several other works have used a reservoir-based computing strategy, as described above. Liquid State Machines, equipped with unsupervised learning models like STDP and BCM (Ivanov and Michmizos, 2021) have shown promising results.

#### 2.1.3. Heterogeneity

Despite the previous works on recurrent spiking neural networks, all these models use a uniform parameter distribution for spiking neuron parameters and their learning dynamics. There has been little research leveraging heterogeneity in the model parameters and their effect on performance and generalization. Recently, Perez-Nieves et al. (2021) introduced heterogeneity in the neuron time constants and showed this improves the model's performance in the classification task and makes the model robust to hyperparameter tuning. She et al. (2021b) also used a similar heterogeneity in the model parameters of a feedforward spiking network and showed it could classify temporal sequences. Again, modeling heterogeneity in the brain cortical networks, Zeldenrust et al. (2021) derived a class of RSNNs that tracks a continuously varying input online.

### 2.2. Action detection using SNNs

SNNs can operate directly on the event data instead of aggregating them, recent works use the concept of time-surfaces (Lagorce et al., 2016; Maro et al., 2020). Escobar et al. (2009) proposed a feed-forward SNN for action recognition using the mean firing rate of every neuron and synchrony between neuronal firing. Yang et al. (2018) used a two-layer spiking neural network to learn human body movement using a gradient descent-based learning the mechanism by encoding the trajectories of the joints as spike trains. Wang W. et al. (2019) proposed a novel Temporal Spiking Recurrent Neural Network (TSRNN) to perform robust action recognition from a video. Using a temporal pooling mechanism, the SNN model provides reliable and sparse frames to the recurrent units. Also, a continuous message passes from spiking signals to RNN helps the recurrent unit retain its long-term memory. The other idea explored in the literature is to capture the temporal features of the input that are extracted by a reservoir network of spiking neurons, the output of which is trained to produce certain desired activities based on some learning rule. Recent research learned video activities with limited examples using this idea of reservoir computing (Panda and Srinivasa, 2018; George et al., 2020; Zhou et al., 2020). We observed that driven/autonomous models are good for temporal dependency modeling of a single-dimensional pre-known time series, but it cannot learn spatio-temporal features together needed for action recognition. Soures and Kudithipudi (2019) used a the deep architecture of a reservoir connected to an unsupervised Winner Take All (WTA) layer, which captures input in a higher dimensional space and encodes that to a low dimensional representation by the WTA layer. All the information from the layers in the deep network is selectively processed using an attention-based neural mechanism. They have used ANN-based spatial feature extraction using ResNet but it is compute-intensive. Some of the recent works also study the effect of heterogeneity in the neuronal parameters (Perez-Nieves et al., 2021; She et al., 2021a). Fang et al. (2021) introduced a learnable leak factor and membrane time constants to introduce heterogeneity in the neurons.

## 3. Methods

### 3.1. Recurrent spiking neural network

SNN consists of spiking neurons connected with synapses. The spiking LIF is defined by the following equations:


(1) $$\begin{array}{l} \tau_{m}\frac{dv}{dt}=a+R_{m}I-v;v=v_{\text{reset}},\text{if }v>v_{\text{threshold}} \end{array}$$

where *R*_*m*_ is membrane resistance, τ_*m*_ = *R*_*m*_*C*_*m*_ is time constant and *C*_*m*_ is membrane capacitance. *a* is the resting potential. *I* is the sum of current from all input synapses connected to the neuron. A spike is generated when membrane potential *v* crosses the threshold, and the neuron enters refractory period *r*, during which the neuron maintains its membrane potential at *v*_reset_. We construct the HRSNN from the baseline recurrent spiking network (RSNN) consisting of three layers: (1) an input encoding layer (I), (2) a recurrent spiking layer (R), and (3) an output decoding layer (O). The recurrent layer consists of excitatory and inhibitory neurons, distributed in a ratio of *N*_*E*_:*N*_*I*_ = 4:1. The PSPs of post-synaptic neurons produced by the excitatory neurons are positive, while those produced by the inhibitory neurons are negative. We used a biologically plausible LIF neuron model and trained the model using STDP rules.

From here on, we refer to connections between I and R neurons as $S_{\mathrm{IR}}$ connections, inter-recurrent layer connections as $S_{\mathrm{RR}}$, and R to O as $S_{\mathrm{RO}}$. We created $S_{\mathrm{RR}}$ connections using probabilities based on Euclidean distance, *D*(*i,j*), between any two neurons *i,j*:


(2) $$\begin{array}{l} P\left(i,j\right)=C\cdot \exp\left(-{\left(\frac{D\left(i,j\right)}{\text{λ}}\right)}^{2}\right) \end{array}$$

with closer neurons having higher connection probability. Parameters *C* and λ set the amplitude and horizontal shift, respectively, of the probability distribution. I contains excitatory encoding neurons, which convert input data into spike trains. $S_{IR}$ only randomly chooses 30% of the excitatory and inhibitory neurons in R as the post-synaptic neuron. The connection probability between the encoding neurons and neurons in the R is defined by a uniform probability $P_{\mathrm{IR}}$, which, together with λ, will be used to encode the architecture of the HRSNN and optimized using BO. In this work, each neuron received projections from some randomly selected neurons in R.

We used unsupervised, local learning to the spiking recurrent model by letting STDP change each $S_{\mathrm{RR}}$ and $S_{\mathrm{IR}}$ connection, modeled as:


(3) $$\begin{array}{l} \frac{dW}{dt}=A_{+}T_{pre}\underset{o}{\sum }\delta \left(t-t_{\text{post}}^{o}\right)-A_{-}T_{\text{post}}\underset{i}{\sum }\delta \left(t-t_{\text{pre}}^{i}\right) \end{array}$$

where *A*_+_, *A*_−_ are the potentiation/depression learning rates and *T*_pre_/*T*_post_ are the pre/post-synaptic trace variables, modeled as,


(4) $$\begin{array}{ll} \tau_{+}^{*}\frac{dT_{\text{pre}}}{dt} & =-T_{\text{pre}}+a_{+}\underset{i}{\sum }\delta \left(t-t_{\text{pre}}^{i}\right) \end{array}$$

(5) $$\begin{array}{ll} \tau_{-}^{*}\frac{dT_{\text{post}}}{dt} & =-T_{\text{post}}+a_{-}\underset{o}{\sum }\delta \left(t-t_{\text{post}}^{o}\right) \end{array}$$

where *a*_+_, *a*_−_ are the discrete contributions of each spike to the trace variable, $\tau_{+}^{*},\tau_{-}^{*}$ are the decay time constants, $t_{\text{pre}}^{i}$ and $t_{\text{post}}^{o}$ are the times of the pre-synaptic and post-synaptic spikes, respectively.

#### 3.1.1. Heterogeneous LIF neurons

The use of multiple timescales in spiking neural networks has several underlying benefits, like increasing the memory capacity of the network. In this paper, we propose the usage of heterogeneous LIF neurons with different membrane time constants and threshold voltages, thereby giving rise to multiple timescales. Due to differential effects of excitatory and inhibitory heterogeneity on the gain and asynchronous state of sparse cortical networks (Carvalho and Buonomano, 2009; Hofer et al., 2011), we use different gamma distributions for both the excitatory and inhibitory LIF neurons. This is also inspired by the brain's biological observations, where the time constants for excitatory neurons are larger than the time constants for the inhibitory neurons. Thus, we incorporate the heterogeneity in our Recurrent Spiking Neural Network by using different membrane time constants τ for each LIF neuron in R. This gives rise to a distribution for the time constants of the LIF neurons in R.

#### 3.1.2. Heterogeneous STDP

Experiments on different brain regions and diverse neuronal types have revealed a wide variety of STDP forms that vary in plasticity direction, temporal dependence, and the involvement of signaling pathways (Sjostrom et al., 2008; Feldman, 2012; Korte and Schmitz, 2016). As described by Pool and Mato (2011), one of the most striking aspects of this plasticity mechanism in synaptic efficacy is that the STDP windows display a great variety of forms in different parts of the nervous system. However, most STDP models used in Spiking Neural Networks are homogeneous with uniform timescale distribution. Thus, we explore the advantages of using heterogeneities in several hyperparameters discussed above. This paper considers heterogeneity in the scaling function constants (*A*_+_, *A*_−_) and the decay time constants (τ_+_, τ_−_).

### 3.2. Classification property of HRSNN

**Figure 1:**
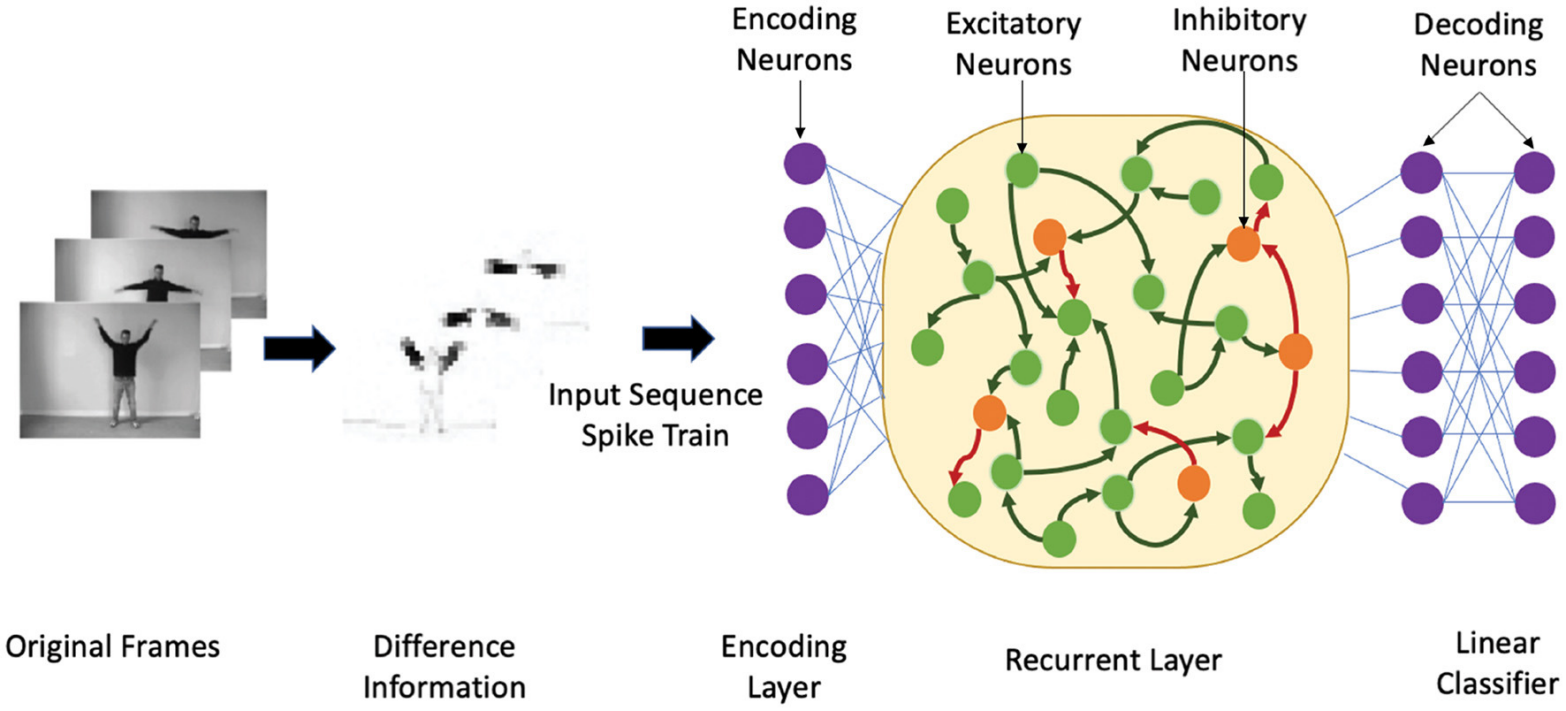
An illustrative example showing the heterogeneous recurrent spiking neural network structure. First, we show the temporal encoding method based on the sensory receptors receiving the difference between two time-adjacent data. Next, the input sequences are encoded by the encoding neurons that inject the spike train into 30% neurons in R.R contains a 4:1 ratio of excitatory (green nodes) and inhibitory (orange nodes), where the neuron parameters are heterogeneous. The synapses are trained using the heterogeneous STDP method.

We theoretically compare the performance of the heterogeneous spiking recurrent model with its homogeneous counterpart using a binary classification problem. The ability of HRSNN to distinguish between many inputs is studied through the lens of the edge-of-chaos dynamics of the spiking recurrent neural network, similar to the case in spiking reservoirs shown by Legenstein and Maass (2007). Also, R possesses a fading memory due to its short-term synaptic plasticity and recurrent connectivity. For each stimulus, the final state of the R, i.e., the state at the end of each stimulus, carries the most information. Figure 1 shows the heterogeneous recurrent spiking neural network model with heterogeneous LIF neurons and heterogeneous STDP synapses used for the classification of spatiotemporal data sequences. The authors showed that the rank of the final state matrix *F* reflects the separation property of a kernel: *F* = [*S*(1) *S*(2) ⋯ *S*(*N*)]^*T*^ where *S*(*i*) is the final state vector of R for the stimulus *i*. Each element of *F* represents one neuron's response to all the *N* stimuli. A higher rank in *F* indicates better kernel separation if all *N* inputs are from *N* distinct classes.

The effective rank is calculated using Singular Value Decomposition (SVD) on *F*, and then taking the number of singular values that contain 99% of the sum in the diagonal matrix as the rank. i.e. *F* = *UΣV*^*T*^ where *U* and *V* are unitary matrices, and Σ is a diagonal matrix diag(λ_1_, λ_2_, λ_3_, …, λ_*N*_) that contains non-negative singular values such that (λ_1_ ≥ λ_2_⋯ ≥ λ_*N*_).

**Definition:** *Linear separation property of a neuronal circuit* C *for* *m* *different inputs* *u*_1_, …, *u*_*m*_(*t*) *is defined as the rank of the* *n*×*m* *matrix* *M* *whose columns are the final circuit states* **x**_*u*_*i*__(*t*_0_) *obtained at time* *t*_0_ *for the preceding input stream* *u*_*i*_.

Following from the definition introduced by Legenstein and Maass (2007), if the rank of the matrix *M* = *m*, then for the inputs *u*_*i*_, any given assignment of target outputs *y*_*i*_ ∈ ℝ at time *t*_0_ can be implemented by C.

We use the rank of the matrix as a measure for the linear separation of a circuit *C* for distinct inputs. This leverages the complexity and diversity of nonlinear operations carried out by *C* on its input to boost the classification performance of a subsequent linear decision-hyperplane.

***Theorem 1:*** *Assuming* $S_{u}$ *is finite and contains* *s* *inputs, let* *r*_Hom_, *r*_Het_ *are the ranks of the* *n*×*s* *matrices consisting of the* *s* *vectors* **x**_*u*_(*t*_0_) *for all inputs* *u* *in* $S_{u}$ *for each of Homogeneous and Heterogeneous RSNNs respectively. Then* *r*_Hom_ ≤ *r*_Het_.

***Short Proof:*** Let us fix some inputs *u*_1_, …, *u*_*r*_ in $S_{u}$ so that the resulting *r* circuit states **x**_*u*_*i*__(*t*_0_) are linearly independent. Using the Eckart-Young-Mirsky theorem for low-rank approximation, we show that the number of linearly independent vectors for HeNHeS is greater than or equal to the number of linearly independent vectors for HoNHoS. The detailed proof is given in the Supplementary material.

**Definition :** *Given* *K*_ρ_ *is the modified Bessel function of the second kind, and* σ^2^, κ, ρ *are the variance, length scale, and smoothness parameters respectively, we define the* ***modified Matern kernel on the Wasserstein metric space*** W *between two distributions* $X,X^{\prime }$ *given as*


(6) $$\begin{array}{l} k\left(X,X^{\prime }\right)=\sigma^{2}\frac{2^{1-\rho }}{\text{Γ}\left(\rho \right)}{\left(\sqrt{2\rho }\frac{W\left(X,X^{\prime }\right)}{\kappa }\right)}^{\rho }H_{\rho }\left(\sqrt{2\rho }\frac{\left(X,X^{\prime }\right)}{\kappa }\right) \end{array}$$

where Γ(.), *H*(.) is the Gamma and Bessel function, respectively.

***Theorem 2:*** *The modified Matern function on the Wasserstein metric space* W *is a valid kernel function*

***Short Proof:*** To show that the above function is a kernel function, we need to prove that Mercer's theorem holds. i.e., (i) the function is symmetric and (ii) in finite input space, the Gram matrix of the kernel function is positive semi-definite. The detailed proof is given in the Supplementary material.

### 3.3. Optimal hyperparameter selection using Bayesian Optimization

While BO is used in various settings, successful applications are often limited to low-dimensional problems, with fewer than twenty dimensions (Frazier, 2018). Thus, using BO for high-dimensional problems remains a significant challenge. In our case of optimizing HRSNN model parameters for 2,000, we need to optimize a huge number of parameters, which is extremely difficult for BO. As discussed by Eriksson and Jankowiak (2021), suitable function priors are especially important for good performance. Thus, we used a biologically inspired initialization of the hyperparameters derived from the human brain (see Supplementary material).

This paper uses a modified BO to estimate parameter distributions for the LIF neurons and the STDP dynamics. To learn the probability distribution of the data, we modify the surrogate model and the acquisition function of the BO to treat the parameter distributions instead of individual variables. This makes our modified BO highly scalable over all the variables (dimensions) used. The loss for the surrogate model's update is calculated using the Wasserstein distance between the parameter distributions.

BO uses a Gaussian process to model the distribution of an objective function and an acquisition function to decide points to evaluate. For data points in a target dataset *x* ∈ *X* and the corresponding label *y* ∈ *Y*, an SNN with network structure V and neuron parameters W acts as a function $f_{V,W}\left(x\right)$ that maps input data *x* to predicted label ỹ. The optimization problem in this work is defined as


(7) $$\begin{array}{l} \underset{V,W}{\min}\underset{x\in X,y\in Y}{\sum }L\left(y,f_{V,W}\left(x\right)\right) \end{array}$$

where V is the set of hyperparameters of the neurons in R (Details of hyperparameters given in the Supplementary material) and W is the multi-variate distribution constituting the distributions of (i) the membrane time constants τ_*m*−*E*_, τ_*m*−*I*_ of the LIF neurons, (ii) the scaling function constants (*A*_+_, *A*_−_) and (iii) the decay time constants τ_+_, τ_−_ for the STDP learning rule in $S_{\mathrm{RR}}$.

Again, BO needs a prior distribution of the objective function $f\left(\vec{x}\right)$ on the given data $D_{1:k}=\left\{{\vec{x}}_{1:k},f\left({\vec{x}}_{1:k}\right)\right\}.$ In GP-based BO, it is assumed that the prior distribution of $f\left({\vec{x}}_{1:k}\right)$ follows the multivariate Gaussian distribution, which follows a Gaussian Process with mean ${\vec{\mu }}_{D_{1:k}}$ and covariance ${\vec{\Sigma }}_{D_{1:k}}$. We estimate ${\vec{\Sigma }}_{D_{1:k}}$ using the modified Matern kernel function, which is given in Equation 6. In this paper, we use *d*(*x, x*′) as the Wasserstein distance between the multivariate distributions of the different parameters. It is to be noted here that for higher-dimensional metric spaces, we use the Sinkhorn distance as a regularized version of the Wasserstein distance to approximate the Wasserstein distance (Feydy et al., 2019).

$D_{1:k}$ are the points that have been evaluated by the objective function, and the GP will estimate the mean ${\vec{\mu }}_{D_{k:n}}$ and variance ${\vec{\sigma }}_{D_{k:n}}$ for the rest unevaluated data $D_{k:n}$. The acquisition function used in this work is the expected improvement (EI) of the prediction fitness as:


(8) $$\begin{array}{l} EI\left({\vec{x}}_{k:n}\right)=\left({\vec{\mu }}_{D_{k:n}}-f\left(x_{\text{best}}\right)\right)\text{Φ}\left(\vec{Z}\right)+{\vec{\sigma }}_{D_{k:n}}\phi \left(\vec{Z}\right) \end{array}$$

where Φ(·) and *ϕ*(·) denote the probability distribution function and the cumulative distribution function of the prior distributions, respectively. $f\left(x_{\text{best}}\right)=\max f\left({\vec{x}}_{1:k}\right)$ is the maximum value that has been evaluated by the original function *f* in all evaluated data $D_{1:k}$ and $\vec{Z}=\frac{{\vec{\mu }}_{D_{k:n}}-f\left(x_{\text{best}}\right)}{{\vec{\sigma }}_{D_{k:n}}}$. BO will choose the data *x*_*j*_= argmax$\left\{EI\left({\vec{x}}_{k:n}\right);x_{j}\subseteq {\vec{x}}_{k:n}\right\}$ as the next point to be evaluated using the original objective function.

## 4. Experiments

### 4.1. Training and inference

**Figure 2:**
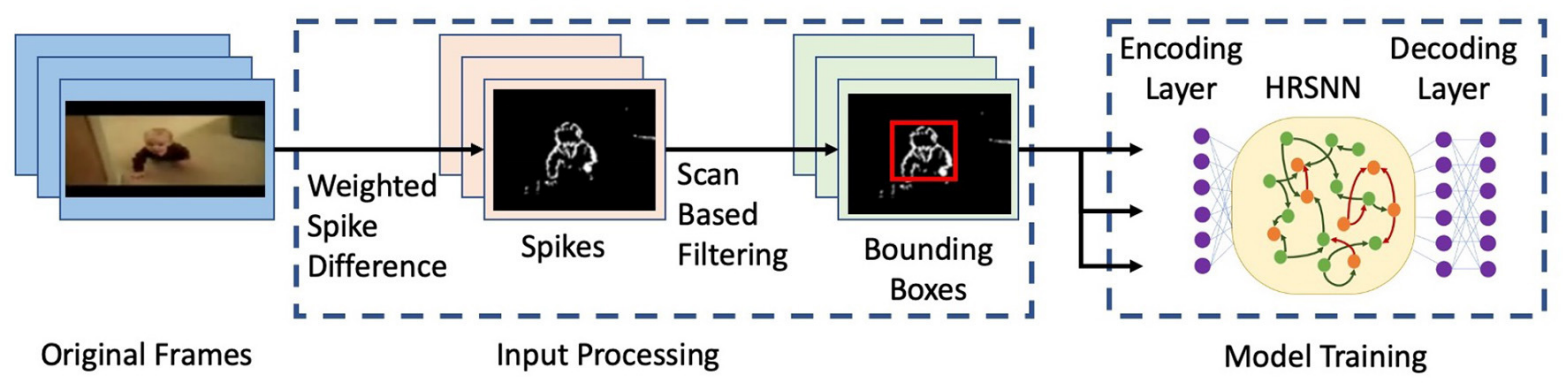
Figure showing a flowchart for the input processing and model training. The figure shows selected frames from a video of the UCF101 dataset (Soomro et al., 2012).

We use a network of leaky integrate and fire (LIF) neurons and train the synapses using a Hebbian plasticity rule called the spike timing dependent plasticity (STDP). The complete network is shown in **Figure 5**. First, to pre-process the spatio-temporal data and remove the background noise which arises due to camera movement and jitters, we use the Scan-based filtering technique as proposed by Panda and Srinivasa (2018) where we create a bounding box and center of gravity of spiking activity for each frame and scan across five directions as shown in Figure 2. Hence, the output of this scan-based filter is fed into the encoding layer, which encodes this information into an array of the spike train. In this paper, we use a temporal coding method. Following Zhou et al. (2020), we use a square cosine encoding method which employs several cosine encoding neurons to convert real-valued variables into spike times. The encoding neurons convert each real value to several spike times within a limited period of encoding time. Each real value is primarily normalized into [0, π], and then converted into spike times as *t*_*s*_ = *T* · cos(*d* + *i* · π/*n*), *d* ∈ [0, π] *i* = 1, 2, …, *n*, where *t*_*s*_ is the spiking time, *T* is the maximum encoding time of each spike, *d* denotes the normalized data, *i* is the sequence number of the encoding neuron, *n* is the number of encoding neurons.

The sensory receptors used for the spatial-temporal data are designed to receive the difference between time-adjacent data in a sequence. The data in each sequence is processed as follows:


(9) $$M_{s}=\left\|\left[\text{Δ}\left(D_{1},D_{2}\right),\ldots ,\text{Δ}\left(D_{N-1},D_{N}\right)\right]\right\|$$

(10) $$\text{Δ}\left(D_{n-1},D_{n}\right)=\{\begin{array}{ll} 1 & \text{ if Δ}\left(D_{n-1},D_{n}\right)\geq \text{ threshold }\cdot \max\left(M_{s}(\cdot )\right)\\ 0 & \text{ else } \end{array}$$

where *M*_*S*_ represents a sequence, and *D*_*n*_ represents an individual data in that sequence. If the difference exceeds the threshold, the encoding neuron will fire at that moment. We use a max-pooling operation before transferring the spike trains to post-synaptic neurons, where each pixel in the output max-pooled frame represents an encoding neuron. This helps in the reduction of the dimensions of the spike train.

The recurrent spiking layer extracts the features of the spatio-temporal data and converts them into linearly separable states in a high-dimensional space. O abstracts the state from R for classification. The state of R is defined as the membrane potential of the output neurons at the end of each spike train converted from the injected spatio-temporal data. After the state is extracted, the membrane potential of the output neuron is set to its initial value. After injecting all sequences into the network, the states of each data are obtained. A linear classifier is employed in this work to evaluate pattern recognition performance. Further details regarding the training and inference procedures are elicited in the Supplementary material.

### 4.2. Baseline ablation models

We use the following baselines for the comparative study:

- **Recurrent Spiking Neural Network with STDP:**
  - Homogeneous LIF Neurons and Homogeneous STDP Learning (**HoNHoS**)
  - Heterogeneity in LIF Neuron Parameters and Homogeneous STDP Learning (**HeNHoS**)
  - Homogeneous LIF Neuron Parameters and Heterogeneity in LTP/LTD dynamics of STDP (**HoNHeS**)
  - Heterogeneity in both LIF and STDP parameters (**HeNHeS**)

- **Recurrent Spiking Neural Network with Backpropagation:**
  - Homogeneous LIF Neurons trained with Backpropagation (**HoNB**)
  - Heterogeneous LIF Neurons trained with Backpropagation (**HeNB**)

## 5. Results

### 5.1. Ablation studies

We compare the performance of the HRSNN model with heterogeneity in the LIF and STDP dynamics (HeNHeS) to the ablation baseline recurrent spiking neural network models described above. We run five iterations for all the baseline cases and show the mean and standard deviation of the prediction accuracy of the network using 2,000 neurons. The results are shown in Table 1. We see that the heterogeneity in the LIF neurons and the LTP/LTD dynamics significantly improve the model's accuracy and error.

**Table 1:** Table comparing the performance of RSNN with homogeneous and heterogeneous LIF neurons using different learning methods with 2,000 neurons.

**Datasets**	**KTH**			**DVS128**		
**Neuron type**	**Homogeneous STDP**	**Heterogeneous STDP**	**Backpropagation**	**Homogeneous STDP**	**Heterogeneous STDP**	**Backpropagation**
Homogeneous LIF	86.33 ± 4.05	91.37 ± 3.15	94.87 ± 2.03	90.33 ± 3.41	93.37 ± 3.05	97.06 ± 2.29
Heterogeneous LIF	92.16 ± 3.17	94.32 ± 1.71	96.84 ± 1.96	92.16 ± 2.97	96.54 ± 1.82	98.12 ± 1.97

### 5.2. Number of neurons

In deep learning, it is an important task to design models with a lesser number of neurons without undergoing degradation in performance. We empirically show that heterogeneity plays a critical role in designing spiking neuron models of smaller sizes. We compare models' performance and convergence rates with fewer neurons in R.

#### 5.2.1. Performance analysis

**Figure 3:**
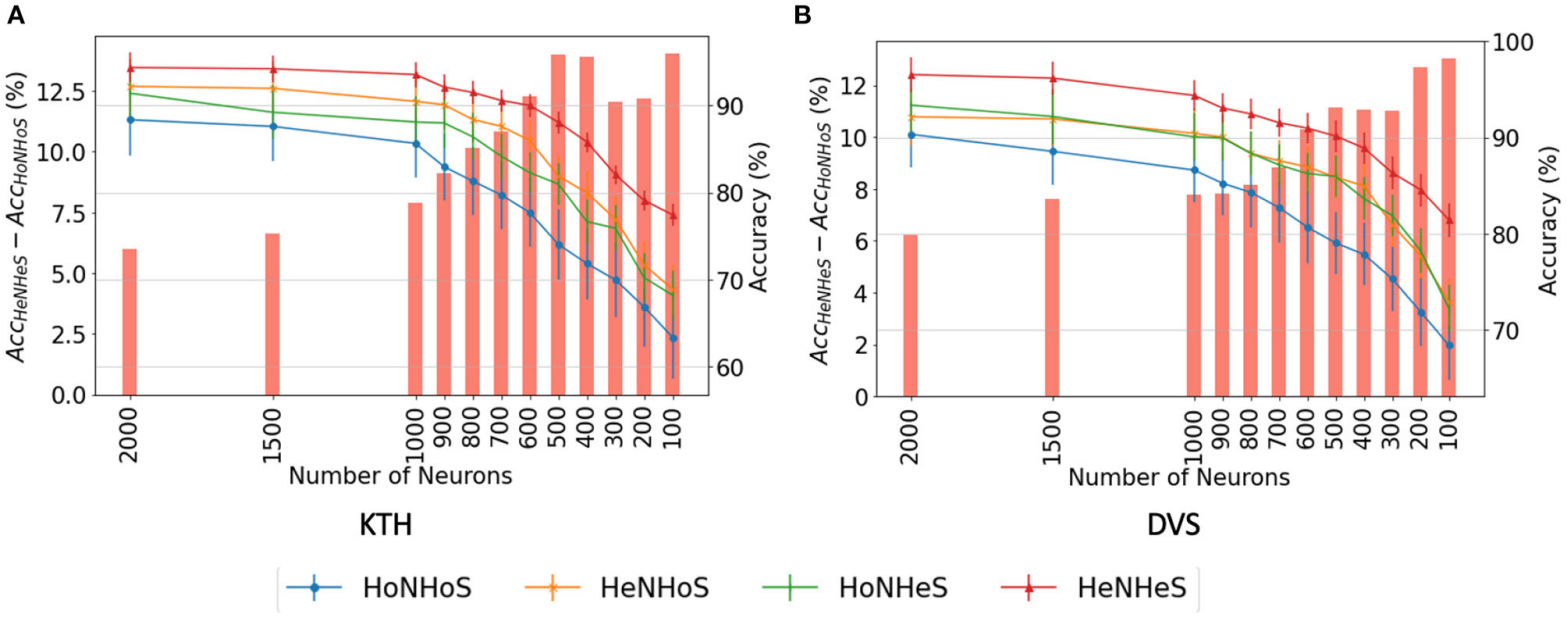
Comparison of performance of HRSNN models for the **(A)** KTH dataset and **(B)** DVS128 dataset for varying number of neurons. The bar graph (left Y-axis) shows the difference between the accuracies between HeNHeS and HoNHoS models. The line graphs (right Y-axis) shows the accuracies (%) for the four ablation networks (HoNHoS, HeNHoS, HoNHeS, and HeNHeS).

We analyze the network performance and error when the number of neurons is decreased from 2,000 to just 100. We report the results obtained using the HoNHoS and HeNHeS models for the KTH and DVS-Gesture datasets. The experiments are repeated five times, and the observed mean and standard deviation of the accuracies are shown in Figure 3. The graphs show that as the number of neurons decreases, the difference in accuracy scores between the homogeneous and the heterogeneous networks increases rapidly.

#### 5.2.2. Convergence analysis with lesser neurons

**Figure 4:**
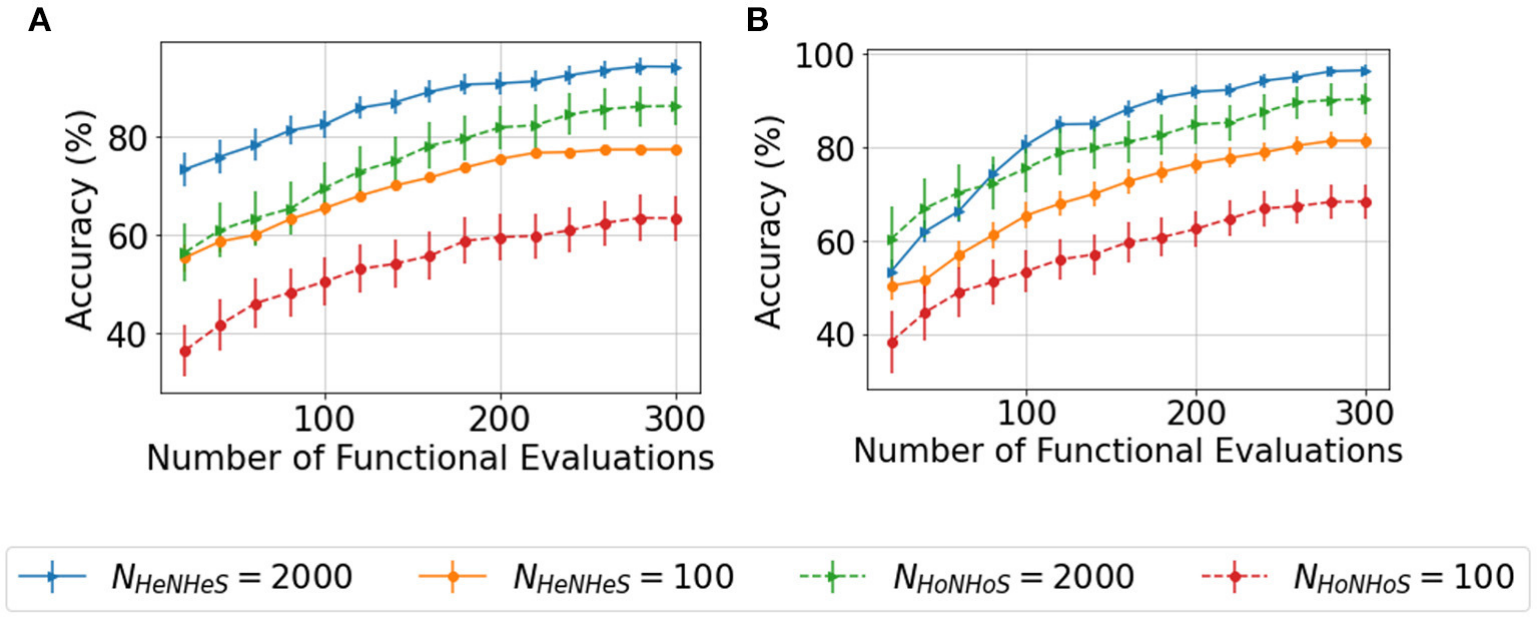
Plots showing comparison of the convergence of the BO with increasing functional evaluations for the **(A)** KTH and **(B)** DVSGesture dataset for varying number of neurons.

Since the complexity of BO increases exponentially on increasing the search space, optimizing the HRSNN becomes increasingly difficult as the number of neurons increases. Thus, we compare the convergence behavior of the HoNHoS and HeNHeS models with 100 and 2,000 neurons each. The results are plotted in Figures 4A, B. Despite the huge number of additional parameters, the convergence behavior of HeNHeS is similar to that of HoNHoS. Also, it must be noted that once converged, the standard deviation of the accuracies for HeNHeS is much lesser than that of HoNHoS, indicating a much more stable model.

### 5.3. Sparse connections

$S_{\mathrm{RR}}$ is generated using a probability dependent on the Euclidean distance between the two neurons, as described by Equation (2), where λ controls the density of the connection, and *C* is a constant depending on the type of the synapses (Zhou et al., 2020).

**Figure 5:**
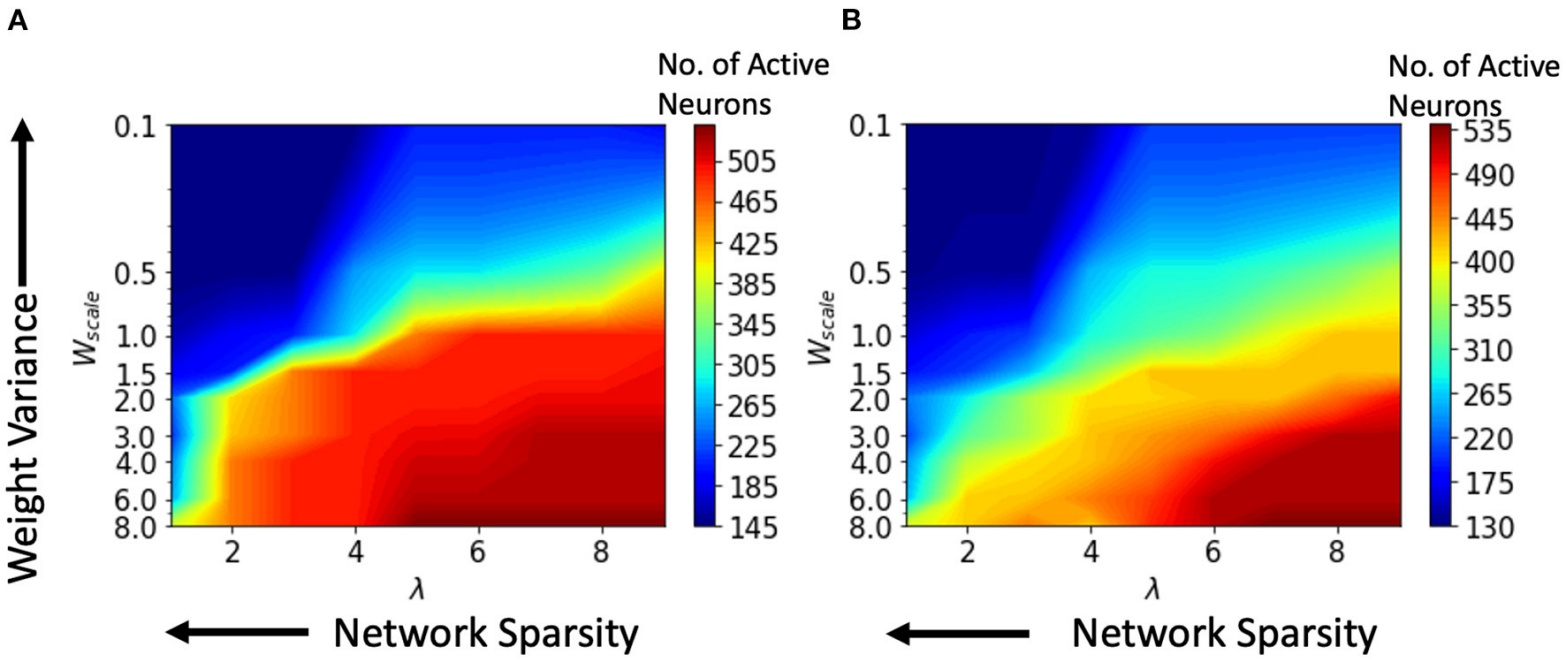
Change in the number of active neurons with network sparsity and weight variance, for **(A)** HoNHoS and **(B)** HeNHeS. The plot is obtained by interpolating 81 points, and each point is calculated by averaging the results from 5 randomly initialized HRSNNs.

We performed various simulations using a range of values for the connection parameter λ and synaptic weight scale *W*_scale_. Increasing λ will increase the number of synapses. Second, the *W*_scale_ parameter determines the mean synaptic strength. Now, a greater *W*_scale_ produces larger weight variance. For a single input video, the number of active neurons was calculated and plotted against the parameter values for synaptic weight *W*_scale_ and network connectivity λ. Active neurons are those that fire at least one spike over the entire test data set. The results for the HoNHoS and HeNHeS are shown in Figures 5A, B, respectively. Each plot in the figure is obtained by interpolating 81 points, and each point is calculated by averaging the results from five randomly initialized with the parameters specified by the point. The horizontal axis showing the increase in λ is plotted on a linear scale, while the vertical axis showing the variation in *W*_scale_ is in a log scale. The figure shows the neurons that have responded to the inputs and reflect the network's activity level. *W*_scale_ is a factor that enhances the signal transmission within R. As discussed by Markram et al. (1997), the synaptic response that is generated by any action potential (AP) in a train is given as *EPSP*_*n*_ = *W*_scale_ × ρ_*n*_ × *u*_*n*_, where ρ_*n*_ is the fraction of the synaptic efficacy for the *n*-th AP and *u*_*n*_ is its utilization of synaptic efficacy. Hence, it is expected that when the *W*_scale_ is large, more neurons will fire. As λ increases, more synaptic connections are created, which opens up more communication channels between the different neurons. As the number of synapses increases, the rank of the final state matrix used to calculate separation property also increases. The rank reaches an optimum for intermediate synapse density, and the number of synapses created increases steadily as λ increases. As λ increases, a larger number of connections creates more dependencies between neurons and decreases the effective separation ranks when the number of connections becomes too large. The results for the variation of the effective ranks with λ and *W*_scale_ are shown in the Supplementary material.

**Figure 6:**
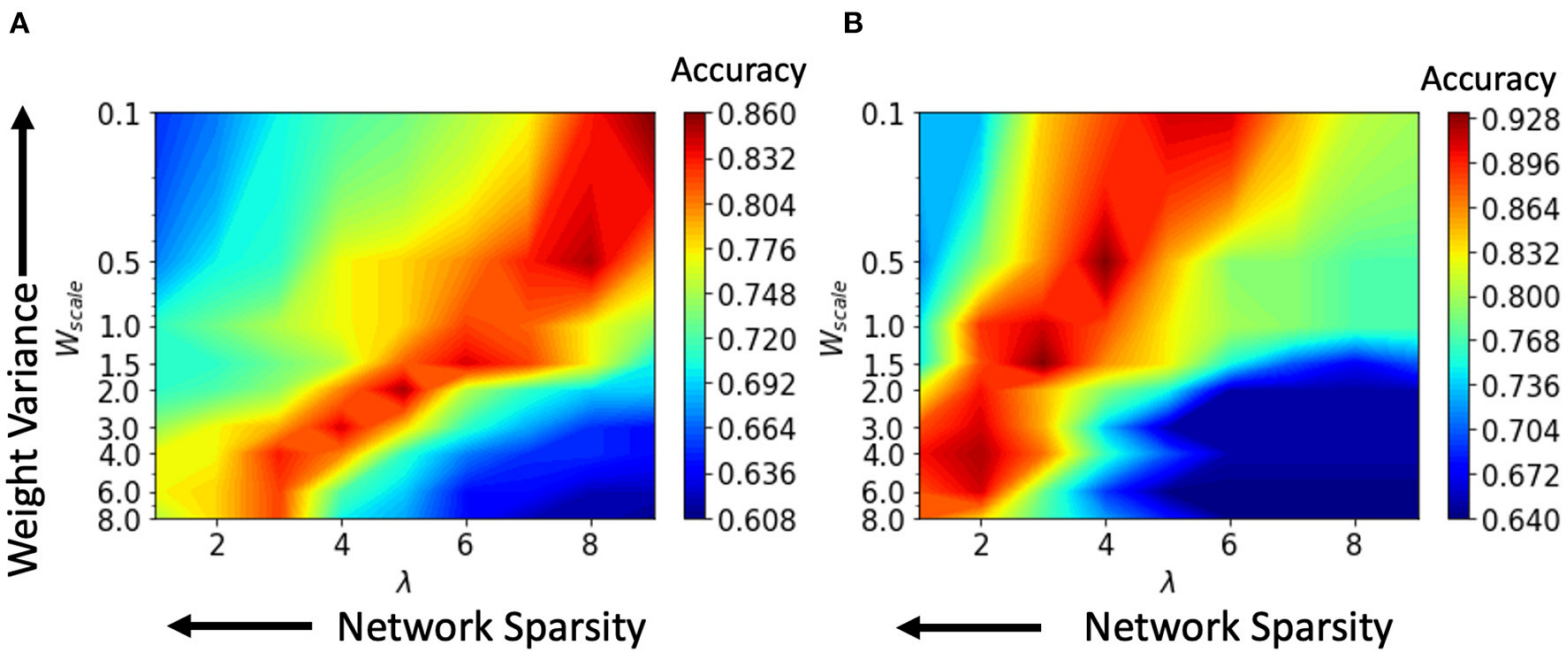
The variation in performance of the action recognition classification task with network sparsity and weight variance for **(A)** HoNHoS and **(B)** HeNHeS. The plot is obtained by interpolating 81 points, and each point is calculated by averaging the results from 5 randomly initialized HRSNNs.

We compare the model's change in performance with varying sparsity levels in the connections and plotted in Figures 6A, B for the HoNHoS and the HeNHeS models. From the figures, we see that for larger values of λ, the performance of both the RSNNs was suboptimal and could not be improved by tuning the parameter *W*_scale_. For a small number of synapses, a larger *W*_scale_ was required to obtain satisfactory performance for HoNHoS compared to the HeNHeS model. Hence, the large variance of the weights leads to better performance. Hence, we see that the best testing accuracy for HeNHeS is achieved with fewer synapses than HoNHoS. It also explains why the highest testing accuracy for the heterogeneous network (Figure 6B) is better than the homogeneous network (Figure 6A), because the red region in Figure 6B corresponds to higher *W*_scale_ values and thus larger weight variance than Figure 6A.

### 5.4. Limited training data

**Figure 7:**
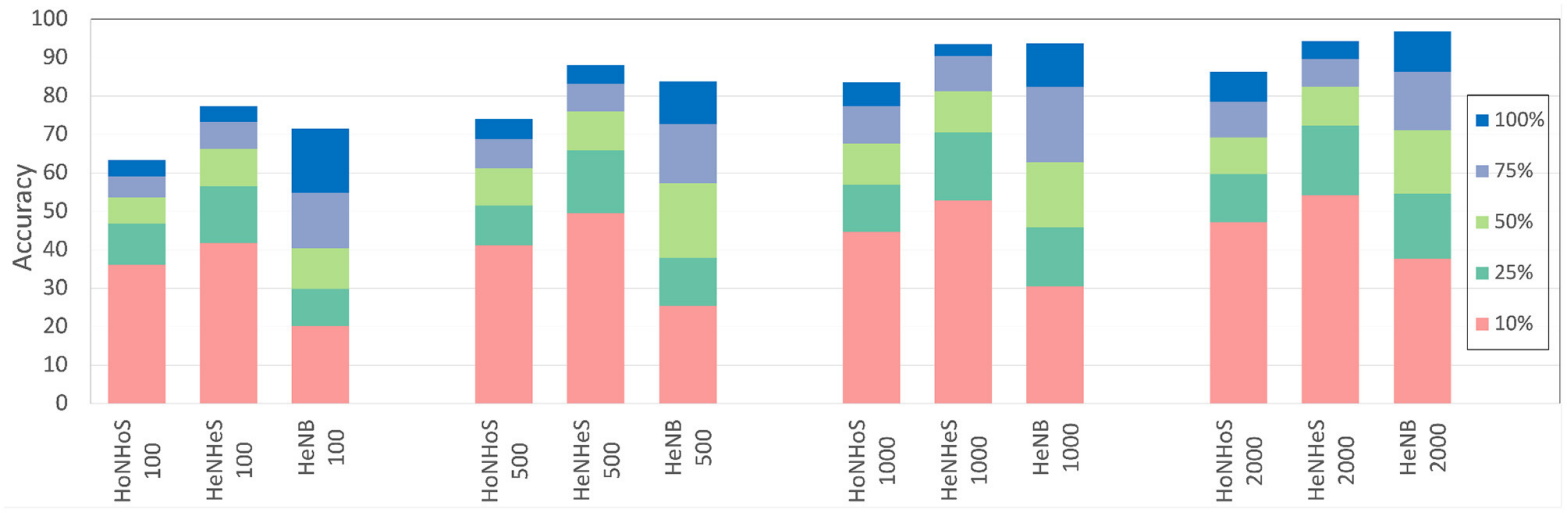
Bar graph showing difference in performance for the different models with increasing training data for the KTH dataset. A similar trend can be observed for the DVS dataset (shown in Supplementary material).

In this section, we compare the performance of the HeNHeS to HoNHoS and HeNB-based spiking recurrent neural networks that are trained with limited training data. The evaluations performed on the KTH dataset are shown in Figure 7 as a stacked bar graph for the differential increments of training data sizes. The figure shows that using 10% training data, HeNHeS models outperform both HoNHoS and HeNB for all the cases. The difference between the HeNHeS and HeNB increases as the number of neurons in the recurrent layer $N_{R}$ decreases. Also, we see that adding heterogeneity improves the model's performance in homogeneous cases. Even when using 2,000 neurons, HeNHeS trained with 10% training data exhibit similar performance to HeNB trained with 25% of training data. It is to be noted here that for the performance evaluations of the cases with 10% training data, the same training was repeated until each model converged.

### 5.5. Comparison with prior work

In this section, we compare our proposed HRSNN model with other baseline architectures. We divide this comparison in two parts as discussed below:

- **DNN-based Models:** We compare the performance and the model complexities of current state-of-the-art DNN-based models (Carreira and Zisserman, 2017; Wang Q. et al., 2019; Bi et al., 2020; Lee et al., 2021; Wang et al., 2021) with our proposed HRSNN models.
- **Backpropagation-based SNN Models:** We compare the performance of backpropagation-based SNN models with HoNB and HeNB-based RSNN models. We observe that backpropagated HRSNN models (HeNB) can achieve similar performance to DNN models but with much lesser model complexity (measured using the number of parameters).
  1. *State-of-the-art BP Homogeneous SNN:* We compare the performance of current state-of-the-art backpropagation-based SNN models (Panda and Srinivasa, 2018; Zheng et al., 2020; Liu et al., 2021; Shen et al., 2021).
  2. *State-of-the-art BP Heterogeneous SNN:* We compare the performances of the current state-of-the-art SNN models, which uses neuronal heterogeneity (Fang et al., 2021; Perez-Nieves et al., 2021; She et al., 2021a). We compare the performances and the model complexities of these models.
  3. *Proposed Heterogeneous Backpropagation Models:* We introduce two new backpropagation-based RSNN models. These models are the Homogeneous Neurons with Backpropagation (HoNB) and the Heterogeneous Neurons with Backpropagation (HeNB). We use our novel Bayesian Optimization to search for the parameters for both of these models.

- **Unsupervised SNN Models:** We also compare the results for some state-of-the-art unsupervised SNN models with our proposed HRSNN models.
  1. *Homogeneous SNN Models:* We compare the performances of some of the state-of-the-art unsupervised SNN models which uses homogeneous neuronal parameters (Meng et al., 2011; Zhou et al., 2020; Ivanov and Michmizos, 2021).
  2. *HRSNN Models:* We compare the above models with respect to our proposed HRSNN models using heterogeneity in both neuronal and synaptic parameters. We compare the model's performance and the model's complexity.

We also compare the average neuronal activation of the homogeneous and the heterogeneous recurrent SNNs for the same given image input for a recurrent spiking network with 2,000 neurons. If we consider the neuronal activation of neuron *i* at time *t* to be ν_*i*_(*t*), the average neuronal activation $\bar{\nu }$ for *T* timesteps is defined as $\bar{\nu }=\frac{\Sigma_{i=0}^{N_{R-1}}\Sigma_{t=0}^{T}\nu_{i}\left(t\right)}{N_{R}}$.

The results obtained are shown in Table 2. The table shows that the heterogeneous HRSNN model has a much lesser average neuronal activation than the homogeneous RSNN and the other unsupervised SNN models. Thus, we conclude that HeNHeS induces sparse activation and sparse coding of information.

**Table 2:** Table showing the comparison of the performance and the model complexities for DNN and supervised and unsupervised SNN models.

	**Model**		**MACs/ACs**	**RGB datasets**			**Event dataset**
				***KTH***	***UCF11***	***UCF101***	***DVS Gesture-128***
**Supervised learning method**							
DNN	PointNet (Wang Q. et al., 2019)		MAC: 152 × 10^9^	-	-	-	95.3
	RG-CNN (Bi et al., 2020)		MAC: 53 × 10^9^	-	-	-	97.2
	I3D (Carreira and Zisserman, 2017)		MAC: 188 × 10^9^	-	90.9	-	96.5
	3D-ResNet-34 (Lee et al., 2021)		MAC: 78.43 × 10^9^	94.78	83.72	-	-
	3D-ResNet-50 (Lee et al., 2021)		MAC: 62.09 × 10^9^	92.31	81.44	-	-
	TDN (Wang et al., 2021)		MAC: 69.67 × 10^9^	99.15	98.03	97.4	-
SNN- supervised (Homogeneous)	STBP-tdBN (Zheng et al., 2020)		AC: 15.13 × 10^7^	-	-	-	96.87
	Shen et al., 2021		AC: 12.14 × 10^7^	-	-	-	98.26
	Liu et al., 2021		AC: 27.59 × 10^7^	90.16	-	-	92.7
	Panda and Srinivasa, 2018		AC: 40.4 × 10^7^	-	-	81.3	-
	HoNB (2,000 Neurons)		AC: 9.54 × 10^7^	94.87	82.89	80.25	97.06
SNN- supervised (Heterogeneous)	Perez-Nieves et al., 2021		AC: 8.94 × 10^7^	-	-	-	82.9
	Fang et al., 2021		AC: 15.32 × 10^7^	-	-	-	97.22
	BPTT (She et al., 2021a)		AC: 13.25 × 10^7^	-	-	-	98.0
	HeNB (2,000 Neurons)		AC: 9.18 × 10^7^	96.84	88.36	84.32	98.12
	**Model**	**Number of neurons**	**MACs/ACs/ Avg. neuron activation (** $\bar{\nu }$ **)**	**RGB datasets**			**Event daset**
				***KTH***	***UCF11***	***UCF101***	**DVS Gesture 128**
**Unsupervised learning method**							
DNN - unsupervised	MetaUVFS (Patravali et al., 2021)	-	MAC: 58.39 × 10^9^	90.14	80.79	76.38	-
	Soomro and Shah, 2017	-	MAC: 63 × 10^9^	84.49	73.38	61.2	-
SNN- unsupervised (Homogeneous)	GRN-BCM (Meng et al., 2011)	1536	$\bar{\nu }=3.56\times 10^{3}$	74.4	-	-	77.19
	LSM STDP (Ivanov and Michmizos, 2021)	135	$\bar{\nu }=10.12\times 10^{3}$	66.7	-	-	67.41
	GP-Assisted CMA-ES (Zhou et al., 2020)	500	$\bar{\nu }=9.23\times 10^{3}$	87.64	-	-	89.25
RSNN-STDP unsupervised (Ours)	HoNHoS	2,000	$\bar{\nu }=3.85\times 10^{3}$	86.33	75.23	74.45	90.33
	HeNHeS	500	$\bar{\nu }=2.93\times 10^{3}$	88.04	71.42	70.16	90.15
	HeNHeS	2,000	$\bar{\nu }=2.74\times 10^{3}$	94.32	79.58	77.33	96.54

Again, comparing state-of-the-art unsupervised learning models for action recognition with our proposed HRSNN models, we see that using heterogeneity in the unsupervised learning models can substantially improve the model's performance while having much lesser model complexity.

## 6. Conclusions

We develop a novel method using recurrent SNN to classify spatio-temporal signals for action recognition tasks using biologically-plausible unsupervised STDP learning. We show how heterogeneity in the neuron parameters and the LTP/LTD dynamics of the STDP learning process can improve the performance and empirically demonstrate their impact on developing smaller models with sparse connections and trained with lesser training data. It is well established in neuroscience that, heterogeneity (De Kloet and Reul, 1987; Shamir and Sompolinsky, 2006; Petitpré et al., 2018) is an intrinsic property of the human brain. Our analysis shows that incorporating such concepts is beneficial for designing high-performance HRSNN for classifying complex spatio-temporal datasets for action recognition tasks.

## Data availability statement

The original contributions presented in the study are included in the article/Supplementary material, further inquiries can be directed to the corresponding author.

## Author contributions

BC developed the main concepts, performed simulation, and wrote the paper under the guidance of SM. All authors assisted in developing the concept and writing the paper. All authors contributed to the article and approved the submitted version.
