# Supplementary material for: Heterogeneous recurrent spiking neural network for spatio-temporal classification
Source: Front Neurosci. 2023 Jan 30;17:994517. doi: 10.3389/fnins.2023.994517 (PMC9922697; doi:10.3389/fnins.2023.994517)
Supplement: Supplementary file 1 [file Data_Sheet_1.pdf]

Supplementary Material

THEOREM 1

Equation for Describing State Dynamics of RSNN We define the state update equation for the recurrent spiking neural network is given as:

$$\mathbf{X}(t+1) = [\mathbf{A} \mid \mathbf{B}] \cdot [\mathbf{X} \mid \mathbf{U}]^{\top}$$
$$[\mathbf{A} \mid \mathbf{B}] = \mathbf{X}(t+1) \cdot \left([\mathbf{X} \mid \mathbf{U}]^{\top}\right)^{\dagger}$$
$$\mathbf{Z} = \mathbf{W} \cdot \mathbf{X} \Rightarrow [\mathbf{A} \mid \mathbf{B}] = \mathbf{Z} \cdot (\mathbf{X})^{\dagger} \tag{S1}$$

For brevity and simplicity, in the rest of the paper, we assume the hidden state h_j^t of a LIF neuron model contains only an activity value v_j^t that evolves according to the equation

$$v_j^{t+1} = \alpha v_j^t + \sum_{i \neq j} \hat{W}_{ji} z_i^t + \sum_i W_{ji}^{\text{in}} x_i^{t+1} - z_j^t v_{\text{th}}$$
$$z_j^t = \sigma\left(v_j^t - v_{\text{th}}\right) \tag{S2}$$

where σ is the nonlinearity (e.g., the Heaviside step function), v_j^t is the activity of neuron j at discrete time t, and v_{th} is the threshold constant. A neuron spikes $\left(z_j^t = 1\right)$ if its activity reaches the activity threshold, and remains silent $\left(z_j^t = 0\right)$ otherwise. W_{ji}^{rec} is a synapse weight from neuron i to neuron j, and α is a constant decay factor. The first term in the above equation models the decay of the activity value over time. The second and third terms model the input of the neuron from other neurons or from the input to the network, respectively. The fourth term $\left(-z_j^t v_{\text{th}}\right)$ ensures that the activity of the neuron drops when it spikes. Hence, we can rewrite Eq.S2 as follows:

$$\left(\alpha \mathbf{x}(t-1) + \sigma\left(\mathbf{W}_{in} F\left[\mathbf{u}(t), \mathbf{x}(t-1)\right] + \boldsymbol{\theta} + \hat{\mathbf{W}} \mathbf{x}(t-1)\right)\right) \tag{S3}$$

For this proof, we consider the HRSNN as a networked dynamical system and follow a similar analysis as done by Tu et al. Tu et al. (2021). Let us consider a networked system consisting of N nodes whose states $x = (x_1, \ldots, x_N)^{\top}$ follow the dynamic equation

$$\frac{dx_i}{dt} = F_i\left(x_i\right) + \sum_j^N A_{ij} G_i\left(x_i, x_j\right) \tag{S4}$$

where $F_i\left(x_i\right)$ is the "local" dynamics at node i (or "self-dynamics") and $G_i\left(x_i, x_j\right)$ is the dynamics expressing the coupling of node i with its neighbors j , according to the adjacency matrix $A \in R^{N \times N}$, representing the interaction network of the system, with A_{ij} capturing the interaction $i \leftarrow j$. Recently, Gao et al.Gao et al. (2016) investigated the resilience of this system in the particular case in which the

functions F and G expressing the self-dynamics and coupling-dynamics are the same at all nodes, i.e., $\forall i, F_i(x_i) = F(x_i)$ and $\forall i, G_i(x_i, x_j) = G(x_i, x_j)$. We define the mean field operator Gao et al. (2016)

$$\mathcal{L}(\mathbf{x}) = \frac{1}{N}\sum_{j=1}^{N} s_j^{\text{out}} x_j / \frac{1}{N}\sum_{j=1}^{N} s_j^{\text{out}} = \frac{\langle \mathrm{s}^{\text{out}} \cdot \mathbf{x}\rangle}{\langle \mathrm{s}^{\text{out}}\rangle}$$

where $\mathrm{s}^{\text{out}} = \left(s_1^{\text{out}}, \ldots, s_N^{\text{out}}\right)$ is the vector of the out-degree of matrix A; then, we characterize the effective state of the networked system using the weighted average node state $x_{eff} = \mathcal{L}(x)$. If the network's degree correlation is low, we can assume that the Hadamard product approximation holds. Then, applying Chebyshev expansion to approximate $F_i(x_i)$ and $G_i(x_i, x_j)$ with polynomial functions of order m and n, respectively, Equation S4 can be reduced to

$$I\left(d_1, \ldots, d_s, x_{\text{eff}}\right) = \frac{dx_{\text{eff}}}{dt} \approx \sum_{s=1}^{s} d_s * x_{\text{eff}}^{s-1} \tag{S5}$$

where $S = \max(m, n), d_s = \left\{ \begin{array}{l} B_{\text{eff}}^s + A_{\text{eff}} * C_{\text{eff}}^s, s \in [1, \min(m, n)] \\ A_{\text{eff}}^s C_{\text{eff}}^s, s \in [m+1, n], m < n \\ B_{\text{eff}}^s, s \in [n+1, m], n < m \end{array} \right.$;

$A_{\text{eff}} = \mathcal{L}\left(s^{in}\right), B_{\text{eff}}^s = \mathcal{L}\left(B^s\right).$, and $C_{\text{eff}}^s = \mathcal{L}\left(C^s\right)$. $B^k = \left(b_{1,k}, \ldots, b_{N,k}\right)^\top$ is the column of the k-th term of the m-order Chebyshev polynomials approximating the self-dynamics $F_i(x_i)$, and $C' = \left(c_{1,l}, \ldots, c_{N,l}\right)^T$ is the column of the l-th factor of the n-order Chebyshev polynomials approximating the coupling-dynamics $G_i(x_i, x_j)$. Therefore, we map the dynamics of Equation S4 into EquationS5 and study the resilience of the system through the behavior of x_{eff} at steady state and its response to a perturbation of one or more of these S parameters. In particular, the conditions for stability of a state x_{eff}^* of the dynamics can thus be associated with a region expressed by the equation set:

$$\left\{ \begin{array}{l} I\left(d_1, \ldots, d_s, x_{\text{eff}}^*\right) = 0 \\ \frac{dl}{dx_{eff}} < 0 \end{array} \right.$$

where the function I represents the system's dynamics and $d_1, \ldots, d_S$ are their control parameters.

Now, for homogeneous and heterogeneous RSNNs, the polynomial approximations using polynomial chaos are derived by Kubota et al. Kubota et al. (2021).

Theorem 1: *Assuming $\mathcal{S}_u$ is finite and contains s inputs, let $r_{\textit{Hom}}, r_{\textit{Het}}$ are the ranks of the $n \times s$ matrices consisting of the s vectors $\mathbf{x}_u(t_0)$ for all inputs u in $\mathcal{S}_u$ for each of Homogeneous and Heterogeneous RSNNs respectively. Then $r_{\textit{Hom}} \leq r_{\textit{Het}}$.*

Proof: To prove that the rank of the Heterogeneous state matrix is greater than the rank of the homogeneous one, we aim to show that the number of linearly independent vectors for HeNHeS is greater than or equal to the number of linearly independent vectors for HoNHoS. However, since the state space of a heterogeneous network is very high-dimensional, we aim to show the results for a low-dimensional projection of this high-dimensional hyperspace. In other words, we aim to show that the number of dimensions of a low-rank approximation of the state-space of the HeNHeS model is greater than the HoNHoS model. For this proof, we treat the HRSNN as a heterogeneous graph and use the network

representation learning framework to embed the network nodes into a low-dimensional vector space by preserving network topology structure, node, and edge information.

First, let us consider that the response of neuron $i \in \mathcal{R}$ is given as

$$\mathbf{y}_i = x^{(1)}\boldsymbol{\beta}_i^1 + \ldots x^{(N)}\boldsymbol{\beta}_i^N + \mathbf{b}_i + \boldsymbol{\epsilon}_i \tag{S6}$$

where $\mathbf{b}_i$ is a constant vector representing a condition-independent mean, and $\boldsymbol{\epsilon}_i$ is noise. The state-space description of the response is represented by a factorization of the vectors $\boldsymbol{\beta}_i^\top = \mathbf{S}^\top \mathbf{w}_i$ where, r is the dimensionality of the subspace for states of the neurons in $\mathcal{R}$. Thus, $\mathbf{w}_i \in \mathbf{R}^r$ is a neuron-specific vector of weights and $\mathbf{S}$ is a matrix of rank r. If $\mathbf{w}_i^\top = \left(\mathbf{w}_i^{1\top}, \ldots, \mathbf{w}_i^\top\right)$, and $\mathbf{S}$ be a block-diagonal matrix given as follows:

$$\mathbf{S} = \begin{pmatrix} \mathbf{S}_1 & & \\ & \ddots & \\ & & \mathbf{S}_P \end{pmatrix} \tag{S7}$$

then we get

$$\mathbf{y}_i = \left(\mathbf{x}^\top \otimes I_T\right) \mathbf{S}^\top \mathbf{w}_i + \mathbf{b}_i + \boldsymbol{\epsilon}_i \tag{S8}$$

If $\mathbf{y}_i$ and $\mathbf{x}$ are the observed response and states of the recurrent neurons, then the collection of all observations for this neuron $\mathbf{y}_i^\top = \left(\mathbf{y}_{i,1}^\top, \ldots, \mathbf{y}_{i,N}^\top\right)$ can be described in terms of all the neuron states in $\mathcal{R}$: $\mathbf{X}_i^\top = (\mathbf{x}_1, \ldots, \mathbf{x}_N)$ by

$$\mathbf{y}_i = (\mathbf{X}_i \otimes I_T)\, \mathbf{S}^\top \mathbf{w}_i + \mathbf{1}_N \otimes \mathbf{b}_i + \boldsymbol{\epsilon}_i \tag{S9}$$

$$= \mathbf{F}_i \mathbf{w}_i + \mathbf{b}_i' + \boldsymbol{\epsilon}_i \tag{S10}$$

where $\mathbf{F}_i = \left(\mathbf{X}_i^\top \otimes I_T\right) \mathbf{S}^\top, \mathbf{b}_i' = \mathbf{1}_N \otimes \mathbf{b}_i$, where $\boldsymbol{\epsilon}_i^\top = \left(\boldsymbol{\epsilon}_{i,1}^\top, \ldots, \boldsymbol{\epsilon}_{i,N}^\top\right)$.

The model's rank corresponds to the rank of $\mathbf{B}$. We first estimate the model parameters with rank $r = 0$ denoting the null model for all elements of $\mathbf{B}$. For the HeNHeS model, the variance of the

Again, let us fix some inputs $u_1, \ldots, u_r$ in S_{univ} so that the resulting r circuit states $\mathbf{x}_{u_i}(t_0)$ are linearly independent. The rather small rank of the state matrix, especially in the ordered regime, can be partly explained by the small number of neurons that get activated (i.e., emit at least one spike) for a given input pattern. For some input pattern u, let the activation vector $\mathbf{x}_u^{\text{act}} \in \{0,1\}^n$ be the vector with the i th entry being 1 if neuron i was activated during the presentation of this pattern. Thus, for HRSNN with HeNHeS, the number of neurons that get activated is higher than in HoNHoS models. Hence $r_{\text{Hom}} \leq r_{\text{Het}}$.

THEOREM 2:

Gaussian processes are used for modeling unknown functions. We study how to extend this model class to model functions in a Wasserstein metric space. We do so in a manner that is both mathematically well-posed and constructive enough to allow the kernel to be computed. This allows the said processes to be trained with standard methods and enables their use in Bayesian optimization of the hyperparameters of the RSNN.

Joint Probability Distribution: We consider the Wasserstein Distance between the joint probability distributions of all the distributions of all the hyperparameters used. The histogram of the hyperparameters, which has heterogeneity in their parameters, shows a distribution of the parameters. For fixed hyperparameters also tuned, we consider a Delta Dirac Distribution at the value of the hyperparameters.

Matern Kernel: One of the most widely-used kernels is the Matérn kernel, which is given by

$$\mathcal{K}\left(x, x'\right) = \sigma^2 \frac{2^{1-\nu}}{\Gamma(\nu)} \left(\sqrt{2\nu}\frac{\|x - x'\|}{\kappa}\right)^{\nu} K_\nu \left(\sqrt{2\nu}\frac{\|x - x'\|}{\kappa}\right)$$

where K_ν is the modified Bessel function of the second kind, and σ^2, κ, ν are the variance, length scale, and smoothness parameters, respectively.

Wasserstein Metric Space:

Let σ and μ be two probability measures on measurable spaces X and Y and their corresponding probability density functions I_0 and $I_1, d\sigma(x) = I_0(x)dx$ and $d\mu(y) = I_1(y)dy$.

Definition The p-Wasserstein distance for $p \in [1, \infty)$ is defined as,

$$W_p(\sigma, \mu) := \left(\inf_{\pi \in \Pi(\sigma,\mu)} \int_{X \times Y} (x - y)^p d\pi(x, y)\right)^{\frac{1}{p}}$$

where $\Pi(\sigma, \mu)$ is the set of all transportation plans, and $\pi \in \Pi(\sigma, \mu)$ such that $\pi(A \times Y) = \sigma(A) \quad$ for any Borel subset $A \subseteq X$ and, $\pi(X \times B) = \mu(B) \quad$ for any Borel subset $B \subseteq Y$. Using Brenier's theorem, for absolutely continuous probability measures σ and μ with respect to Lebesgue measure, the p-Wasserstein distance can be derived as,

$$W_p(\sigma, \mu) = \left(\inf_{f \in MP(\sigma,\mu)} \int_X (f(x) - x)^p d\sigma(x)\right)^{\frac{1}{p}}$$

where, $MP(\sigma, \mu) = \left\{f : X \to Y \mid f_{\#}\sigma = \mu\right\}$ and $f_{\#}\sigma$ represents the pushforward of measure σ and is characterized as, $\int_{f^{-1}(A)} d\sigma = \int_A d\mu$ for any Borel subset $A \subseteq Y$

Sliced Wasserstein Distance: We use the sliced Wasserstein distance to represent the family of one-dimensional distributions for the higher-dimensional probability distribution and then calculate the distance between two input higher-dimensional distributions as a functional on the Wasserstein distance of their one-dimensional representations. In this sense, the distance is obtained by solving several one-dimensional optimal transport problems with closed-form solutions.

Definition Let σ and μ be two continuous probability measures on $\mathbb{R}^d$ with corresponding positive probability density functions I_1 and I_0. The Sliced Wasserstein distance between μ and σ is defined as,

$$\begin{aligned} W_S(\mu, \sigma) &:= \left(\int_{\mathbb{S}^{d-1}} W_2^2\left(\mathcal{S}I_1(., \theta), \mathcal{S}I_0(., \theta)\right) d\theta\right)^{\frac{1}{2}} \\ &= \left(\int_{\mathbb{S}^{d-1}} \int_{\mathbb{R}} \left(f_\theta(t) - t\right)^2 \mathcal{S}I_0(t, \theta) dt d\theta\right)^{\frac{1}{2}} \end{aligned}$$

where f_θ is the MP map between $\mathcal{S}I_0(.,\theta)$ and $\mathcal{S}I_1(.,\theta)$ such that,

$$\int_{-\infty}^{f_\theta(t)} \mathcal{S}I_1(\tau,\theta)d\tau = \int_{-\infty}^{t} \mathcal{S}I_0(\tau,\theta)d\tau, \forall\theta \in \mathbb{S}^{d-1}$$

or equivalently in the differential form,

$$\frac{\partial f_\theta(t)}{\partial t}\mathcal{S}I_1\left(f_\theta(t),\theta\right) = \mathcal{S}I_0(t,\theta), \quad \forall\theta \in \mathbb{S}^{d-1}.$$

Positive Definite: A positive definite (PD) (resp. conditional negative definite) kernel on a set M is a symmetric function $\mathcal{K}: M \times M \to \mathbb{R}, \mathcal{K}(I_i, I_j) = \mathcal{K}(I_j, I_i)$ for all $I_i, I_j \in M$, such that for any $n \in N$, any elements $I_1, \ldots, I_n \in X$, and numbers $c_1, \ldots, c_n \in \mathbb{R}$, we have

$$\sum_{i=1}^{n}\sum_{j=1}^{n} c_i c_j \mathcal{K}(I_i, I_j) \geq 0 \quad (\text{ resp. } \leq 0)$$

with the additional constraint of $\sum_{i=1}^{n} c_i = 0$ for the conditionally negative definiteness.

We start by proving that for one-dimensional probability density functions, the 2-Wasserstein Matern kernel is a positive definite kernel. We first demonstrate that the Sliced Wasserstein Matern kernel of probability measures is a positive definite kernel. We proceed with our argument by showing that there is an explicit formulation for the nonlinear mapping to the kernel space and define a family of kernels based on this mapping.

First, we start by proving that for one-dimensional probability density functions, the 2-Wasserstein Matern kernel is a positive definite kernel.

Theorem : *Let M be the set of absolutely continuous one-dimensional positive probability density functions and define $\mathcal{K}: M \times M \to \mathbb{R}$ to be $\mathcal{K}(I_i, I_j) := \sigma^2 \frac{2^{1-\nu}}{\Gamma(\nu)}\left(\sqrt{2\nu}\frac{W_2(I_i,I_j)}{\kappa}\right)^{\nu} K_\nu\left(\sqrt{2\nu}\frac{W_2(I_i,I_j)}{\kappa}\right)$, then $\mathcal{K}(.,.)$ is a positive definite kernel for all $\gamma > 0$.*

Here, K_ν is the modified Bessel function of the second kind, and σ^2, κ, ν are the variance, length scale, and smoothness parameters, respectively.

Proof: In order to be able to show this, we first show that for absolutely continuous one-dimensional positive probability density functions there exists an inner product space $\mathcal{V}$ and a function $\psi: M \to \mathcal{V}$ such that $W_2(I_i, I_j) = \|\psi(I_i) - \psi(I_j)\|_{\mathcal{V}}$

Let σ, μ, and ν be probability measures on $\mathbb{R}$ with corresponding absolutely continuous positive density functions I_0, I_1, and I_2. Let $f, g, h: \mathbb{R} \to \mathbb{R}$ be transport maps such that $f_{\#}\sigma = \mu, g_{\#}\sigma = \nu$, and $h_{\#}\mu = \nu$. In the differential form this is equivalent to $f' I_1(f) = g' I_2(g) = I_0$ and $h' I_2(h) = I_1$ where

$I_1(f)$ represents $I_1 \circ f$. Then we have,

$$W_2(I_1, I_0) = \int_{\mathbb{R}} (f(x) - x) I_0(x) dx$$
$$W_2(I_2, I_0) = \int_{\mathbb{R}} (g(x) - x) I_0(x) dx$$
$$W_2(I_2, I_1) = \int_{\mathbb{R}} (h(x) - x) I_1(x) dx$$

We follow the work of Wang et al. [45] and Park et al. [31] and define a nonlinear map with respect to a fixed probability measure, σ with corresponding density I_0, that maps an input probability density to a linear functional on the corresponding transport map. More precisely, $\psi_\sigma(I_1(.)) := (f(.) - id(.))\sqrt{I_0(.)}$ where $id(.)$ is the identity map and $f' I_1(f) = I_0$. Notice that such ψ_σ maps the fixed probability density I_0 to zero, $\psi_\sigma(I_0(.)) = (id(.) - id(.))\sqrt{I_0(.)} = 0$ and it satisfies,

$$W_2(I_1, I_0) = \|\psi_\sigma(I_1)\|_2$$
$$W_2(I_2, I_0) = \|\psi_\sigma(I_2)\|_2$$

More importantly, we demonstrate that $W_2(I_2, I_1) = \|\psi_\sigma(I_1) - \psi_\sigma(I_2)\|_2$. To show this, we can write,

$$\begin{aligned}
W_2(I_2, I_1) &= \int_{\mathbb{R}} (h(x) - x) I_1(x) dx \\
&= \int_{\mathbb{R}} (h(f(\tau)) - f(\tau)) f'(\tau) I_1(f(\tau)) d\tau \\
&= \int_{\mathbb{R}} (g(\tau) - f(\tau)) I_0(\tau) d\tau \\
&= \int_{\mathbb{R}} ((g(\tau) - \tau) - (f(\tau) - \tau)) I_0(\tau) d\tau \\
&= \|\psi_\sigma(I_1) - \psi_\sigma(I_2)\|_2
\end{aligned}$$

Finally, we know that the one-dimensional transport maps are unique, therefore if $(h \circ f)\#\sigma = \nu$ and $g_{\#}\sigma = \nu$ then $h \circ f = g$.

We showed that there exists a nonlinear map $\psi_\sigma : M \to \mathcal{V}$ for which $W_2(I_i, I_j) = \|\psi_\sigma(I_i) - \psi_\sigma(I_j)\|_2$ and as shown by Jayasumana et al. Jayasumana et al. (2015) and Kolouri et al. Kolouri et al. (2016), we can conclude that, $\mathcal{K}(I_i, I_j)$ is a positive definite kernel.

Theorem 2: *The modified Matern function on the Wasserstein metric space $\mathcal{W}$ is a valid kernel function*

Proof: To show that the above function is a kernel function, we need to prove that Mercer's theorem holds. i.e., (i) the function is symmetric and (ii) in finite input space, the Gram matrix of the kernel function is positive semi-definite. The Sliced Wasserstein distance, as defined above, is symmetric, and it satisfies subadditivity and coincidence axioms; hence it is a true metric. Kolouri et al. (2015).

First note that for an absolutely continuous positive probability density function, $I \in M$, each hyperplane integral, $\mathcal{S}I(., \theta), \forall \theta \in \mathbb{S}^{d-1}$ is a one dimensional absolutely continuous positive probability density

function. Therefore,

$$\sum_{i=1}^{N}\sum_{j=1}^{N} c_i c_j W_2^2\left(\mathcal{S}I_i(.,\theta), \mathcal{S}I_j(.,\theta)\right) \leq 0, \forall \theta \in \mathbb{S}^{d-1}$$

where $\sum_{i=1}^{N} c_i = 0$. Integrating the left-hand side of the above inequality over θ leads to,

$$\int_{\mathbb{S}^{d-1}} \left(\sum_{i=1}^{N}\sum_{j=1}^{N} c_i c_j W_2^2\left(\mathcal{S}I_i(.,\theta), \mathcal{S}I_j(.,\theta)\right) d\theta\right) \leq 0 \Rightarrow$$

$$\sum_{i=1}^{N}\sum_{j=1}^{N} c_i c_j \left(\int_{\mathbb{S}^{d-1}} W_2^2\left(\mathcal{S}I_i(.,\theta), \mathcal{S}I_j(.,\theta)\right) d\theta\right) \leq 0 \Rightarrow$$

$$\sum_{i=1}^{N}\sum_{j=1}^{N} c_i c_j W_{\mathcal{S}}^2\left(I_i, I_j\right) \leq 0$$

Therefore $W_{\mathcal{S}}^2(.,.)$ is conditionally negative definite, and hence from the previous theorem, we have that $\mathcal{K}\left(I_i, I_j\right)$ is a positive definite kernel for $\gamma > 0$.

BAYESIAN OPTIMIZATION

Brain-Inspired Initialization

Mejias et al. Mejias and Longtin (2014) showed that in real cortical populations, excitatory and inhibitory subpopulations of neurons exhibit different cell-to-cell heterogeneities for each type of subpopulation in the system. The authors discussed the highly differentiated roles for heterogeneity, depending on excitatory or inhibitory neuron subpopulation. For example, heterogeneity among excitatory neurons non-linearly increases the mean firing rate and linearizes the f-I curves, while heterogeneity among inhibitory neurons decreases the network activity level and induces divisive gain effects in the f-I curves of the excitatory cells, providing an effective gain control mechanism to influence information flow. We use the Allen human brain-based initialization using separate distributions for the excitatory and inhibitory neuron populations. A gamma distribution is fitted using the Kernel Density Estimation Method on the data for the membrane timescales, which is used to sample the values of all the membrane time constants of the recurrent neurons in the HRSNN model. The fitted distribution is shown in Fig. S1

Initialization Distributions

**Notation**	**Full Form**	**Notation**	**Full Form**
**SNN**	Spiking Neural Network	$\mathcal{I}$	Input Layer
**RSNN**	Recurrent SNN	$\mathcal{R}$	Recurrent Layer
**HRSNN**	Heterogeneous RSNN	$\mathcal{O}$	Output Layer
**STDP**	Spike Timing Dependent Plasticity	$\mathcal{S}_{\mathbf{XY}}$	Connections between layers X and Y
**LIF**	Leaky Integrate and Fire	N	Number of neurons
**HoNHoS**	Homogeneous LIF, Homogeneous STDP	**HeNHeS**	Heterogeneous LIF, Heterogeneous STDP
**HeNHeS**	Heterogeneous LIF, Homogeneous STDP	**HoNHeS**	Homogeneous LIF, Heterogeneous STDP

Table S1: Table showing the notations used in the paper

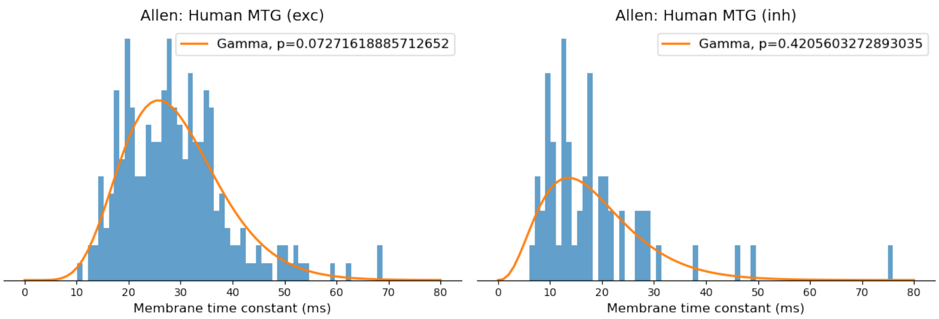

Figure S1: The fitted gamma distribution to the Allen Human brain atlas-based distribution for membrane time constants

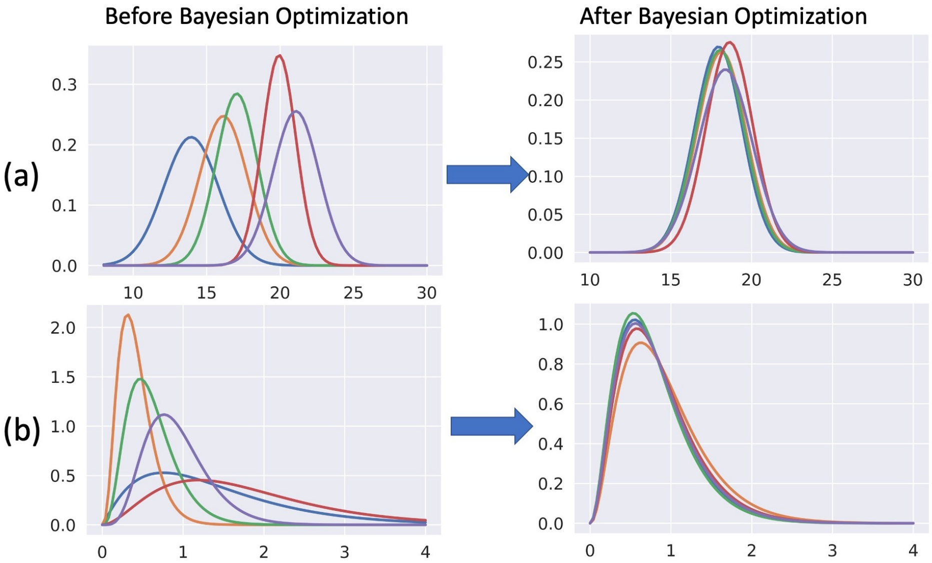

Figure S2: Figure (a) shows the initialization and the final distributions of τ_+ (STDP Parameter). We can observe a similar behavior on optimizing τ_-, $\eta_\pm$. Fig. (b) shows the initialization and the final distributions of $\tau_m^{(e)}$ (Excitatory Membrane Potential of LIF Neuron). We can observe a similar behavior on optimizing $\tau_m^{(i)}$

We plot the initial and the final distributions for Bayesian Optimization. We randomly initialize the distribution parameters within some certain range for each iteration of the Bayesian Optimization. An example initialization of normal distributions for τ_+ of the STDP parameters and the final distributions for each of the 5 cases are shown in Fig. S2(a). Similarly, Fig. S2(b) shows the 5 random initial and the subsequent final distributions output of the Bayesian Optimization for $\tau_m^{(e)}$. Similar results can be seen for the other hyperparameters which are optimized. The average of the parameters for the final optimal distributions are summarized in Table S3.

Hyperparameters Optimized

The list of the hyperparameters optimized using the Bayesian Optimization technique is shown in Table S2. We also show the average parameter values of the distributions searched using the Bayesian Optimization process. The results are shown in Table S3 and illustrated in Fig. S2, as discussed before. It must be noted here that for simplicity, we consider a simple distribution (normal or gamma) for the

Parameter	Initial Value	Range
η	10	(0,50)
γ	5	(0,10)
ζ	2.5	(0,10)
η^*	1	(0,3)
g	2	(0,10)
ω	0.5	(0,1)
k	50	(0,100)
λ (KTH, DVS)	1	(0,2)
λ (UCF)	1.5	(0,4)
P_{IR}	0.05	(0,0.1)
τ_{n-E}, τ_{n-I} (KTH, DVS)	$50ms$	$(0ms, 100ms)$
τ_{n-E}, τ_{n-I} (UCF)	$100ms$	$(0ms, 300ms)$
$A_{en-R}, A_{EE}, A_{EI}, A_{IE}, A_{II}$	30	(0,60)

Table S2: The list of parameter settings for the Bayesian Optimization-based hyperparameter search

	Parameter	Distribution	
STDP Parameter	τ_+	Normal	$\bar{\mu} = 18.235$ $\bar{\sigma} = 1.522$
	τ_-	Normal	$\bar{\mu} = 22.382$ $\bar{\sigma} = 1.768$
	η_+	Normal	$\bar{\mu} = 0.516$ $\bar{\sigma} = 0.0055$
	η_-	Normal	$\bar{\mu} = 0.448$ $\bar{\sigma} = 0.0057$
LIF Parameter	$\tau_m^{(e)}$	Gamma	$\bar{\alpha} = 2.89$ $1/\bar{\beta} = 0.248$
	$\tau_m^{(i)}$	Gamma	$\bar{\alpha} = 5.14$ $1/\bar{\beta} = 0.313$

Table S3: Table showing the average final distributions of the hyperparameters

hyperparameter optimization in this paper. In reality, for optimal optimization performance, one might use a non-parametric distribution which might be a good future work for this paper.

VARIATION OF RANK WITH SPARSITY AND WEIGHT SCALE

Here, we show the variation of the rank with the network sparsity factor λ and the synaptic weight scale factor W_{scale}. The figure is shown in Fig. S3. From the figure we observe the variation of the rank of the matrix with the network sparsity. This also supports our intial claim that the rank of the final state matrix can be used as a measure for the linear separation property of the HRSNN model. Comparing Figs.S3(a) and (b) we also see that the performance of the model is the highest near the regions between the chaos and order. This is built on the works done by Legenstein et al. Legenstein and Maass (2007).

GENERALIZATION AND OVERFITTING

To study the generalizability of the HRSNN models, we look into the difference between the training and testing accuracies of the models. We see that heterogeneity in just LIF neurons has the worst generalization ability despite giving good test accuracy scores. On the other hand, heterogeneity in STDP parameters

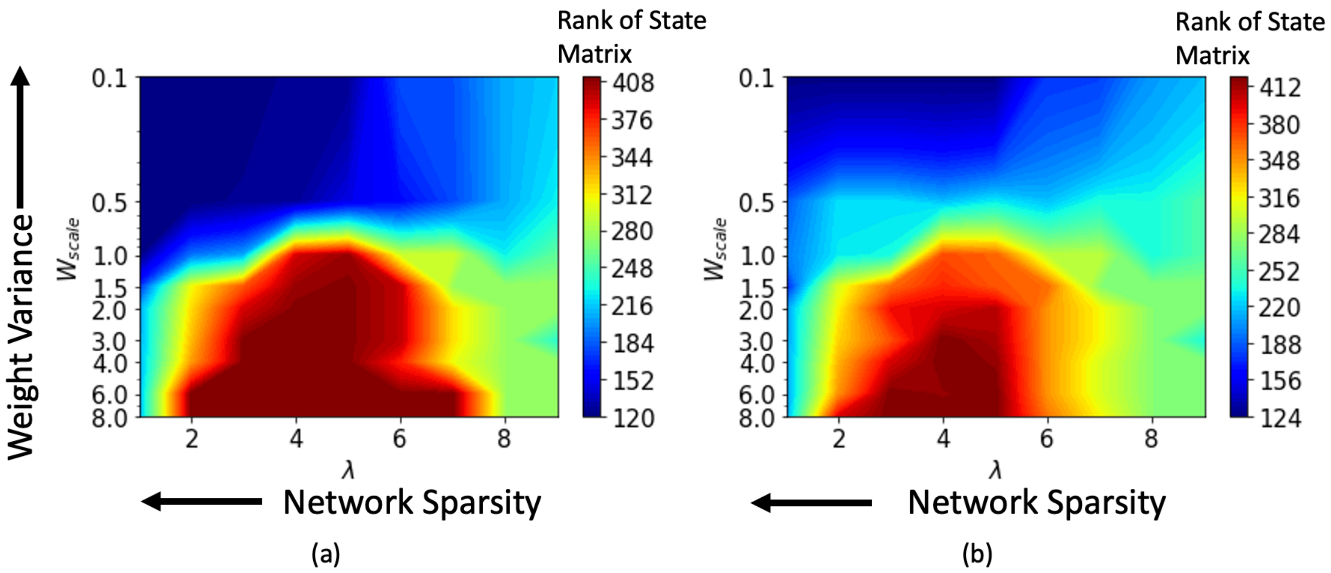

Figure S3: Change in the effective ranks of the final state matrix of HRSNN with 2000 neurons with network sparsity λ and weight variance W_{scale}, for (a)HoNHoS and (b)HeNHeS. The plot is obtained by interpolating 81 points, and each point is calculated by averaging the results from 5 randomly initialized HRSNNs.

shows the best generalization results. HeNHeS model shows the best case where we have good testing accuracy and good generalization error. Hence, these results prove that heterogeneity in both LIF neurons and STDP parameters is needed for a model to perform well.

RESULTS WITH LIMITED TRAINING DATA

In this section, we plot the stacked bar graph for the results obtained from the DVS gesture dataset trained with limited training data. The results show a similar trend to the KTH dataset results shown in the paper.

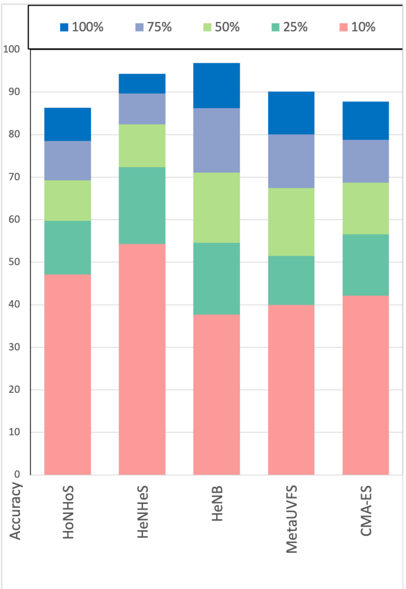

Figure S4: Figure showing the input processing and model training for the different models

Neurons	Models	KTH			DVS Gesture 128		
		Training Accuracy	Testing Accuracy	Generalization Error	Training Accuracy	Testing Accuracy	Generalization Error
100	HoNHoS	75.94	63.38	12.56	87.83	68.38	19.45
	HeNHoS	88.34	68.89	19.45	97.24	72.89	24.35
	HoNHeS	78.78	68.22	10.56	87.67	72.22	15.45
	HeNHeS	88.92	77.43	11.49	98.4	81.43	16.97
2000	HoNHoS	94.1	88.33	5.77	96.16	90.33	5.83
	HeNHoS	98.69	92.16	6.53	99.07	92.16	6.91
	HoNHeS	95.4	91.37	4.03	96.5	93.37	3.13
	HeNHeS	98.85	94.32	4.53	99.75	96.54	3.21

Table S4: Table showing the generalization performance of the ablation HRSNN and MRSNN models.

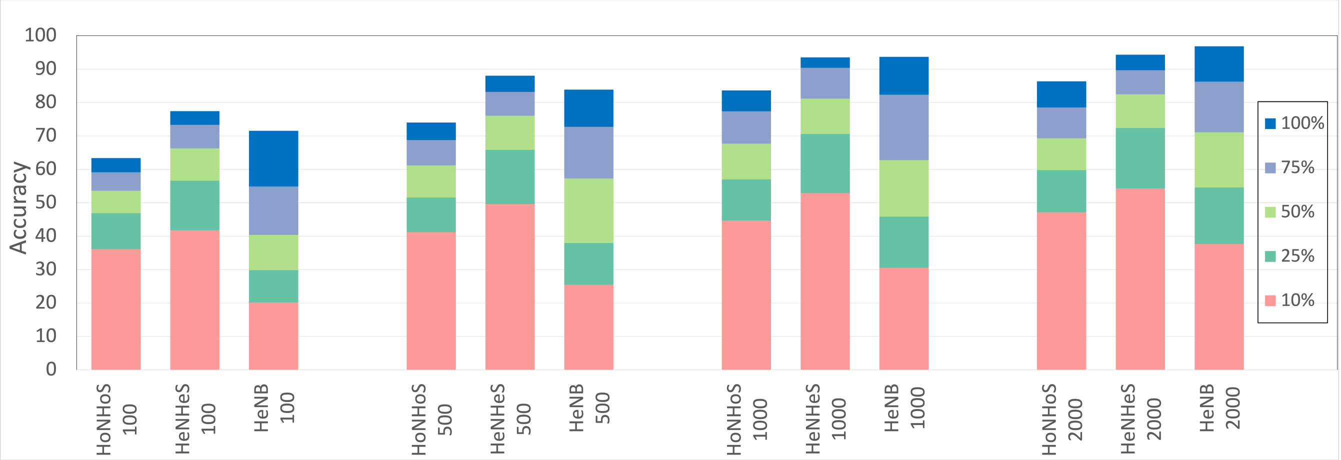

Figure S5: Bar graph showing the difference in performance for the different models with increasing training data for the DVS dataset.

CONFUSION MATRIX

We present an example confusion matrix for each KTH, UCF11, and the DVS128 dataset. We see that in the KTH dataset, the model struggles the most in the classification of *jogging*, where it is mostly misclassified as *running*. A similar trend for *running* could be observed, which was sometimes confused by the model as *jogging*. In the UCF11 dataset, the model performed the poorest in classifying *basketball* and *walking dog*. For the first case, the model confused *basketball* with *tennis* the most. For the second case, the model mistook it for *biking* and *horse riding* the most number of times. We also see the model performs much better on the DVS128 Gesture dataset, where the maximum error source was attributed to the misclassification of *hand clap* as *air drums*.

COMPUTATIONAL COST:

To estimate the efficiency of BP-SNNs and compare them with DNNs, we calculate the number of computations required in terms of accumulation (AC) and multiply-accumulation (MAC) operations Wong et al. (2020). In DNNs, the contribution from one neuron to another requires a MAC for every timestep, where each input activation is multiplied by the respective weight before it is added to the internal sum. On the other hand, for a spiking neuron, a transmitted spike requires only an accumulation at the target neuron, adding weight to the potential, where spikes may be quite sparse. As it is much more energetically expensive to calculate MACs than ACs (on a 45nm 0.9V chip, a 32-bit floating-point (FL) MAC operation consumes 4.6 pJ and 0.9 pJ for an AC operation Chakraborty et al. (2021), Panda and Srinivasa (2018)), the relative efficiency of SNNs is determined by the number of connections multiplied by activity sparsity and the spiking neuron model complexity.

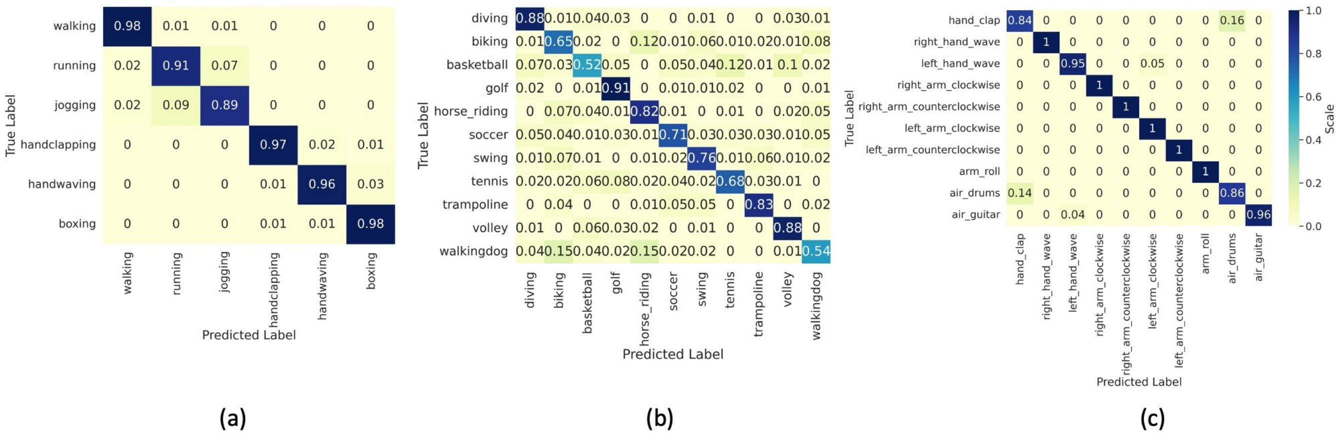

Figure S6: Figure showing the confusion matrices for (a) KTH dataset (b) UCF11 dataset and (c)DVS128 Gesture dataset

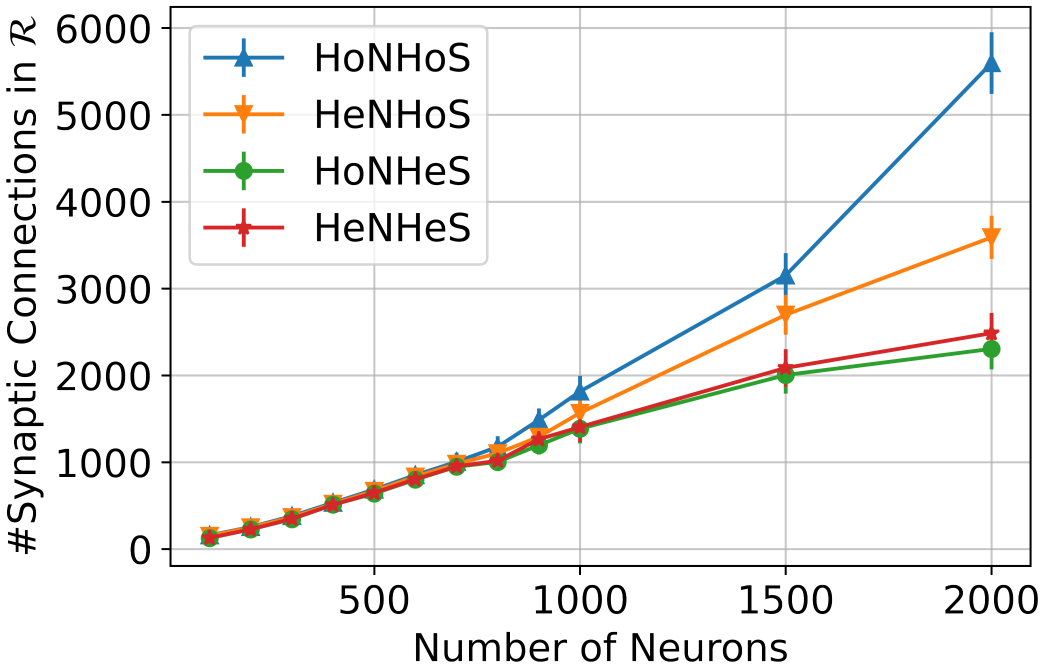

Figure S7: Figure showing the increase in the number of parameters with the increase in the number of neurons

NUMBER OF SYNAPTIC CONNECTIONS

We plot the number of synaptic connections of the recurrent layer in the HRSNN/MRSNN models as a function of the number of neurons. The number of synaptic connections is enumerated for the network with the least sparsity λ, keeping the weight scaling factor $W_{scale} = 1$. The hypothesis is that results can be observed on fixing other constants for W_{scale}. We know that increasing the number of synaptic connections exponentially increases the number of trainable parameters of the network. We plot the observed number of synaptic connections in the RSNN model with respect to the number of neurons in the recurrent layer. The results are plotted in Fig. S7. We see that for HoNHoS, the number of synaptic connections increases exponentially. However, introducing heterogeneity (either in neuronal or synaptic dynamics) helps us

decrease the number of synaptic connections. We observe that the number of synaptic connections for the HoNHoS model increases exponentially. It might be interpreted as if we aim to generate a complete graph, we model this as a random graph process. As shown by Frieze et al. Frieze and Karoński (2016), for a network of size N, the expected number of edges to get a complete graph is given by $\mathcal{O}(\frac{N}{2}\log N)$. HoNHoS models follow this complexity order. However, introducing heterogeneity significantly decreases the required number of synaptic connections.

REFERENCES

Chakraborty, B., She, X., and Mukhopadhyay, S. (2021). A fully spiking hybrid neural network for energy-efficient object detection. *IEEE Transactions on Image Processing* 30, 9014–9029

Frieze, A. and Karoński, M. (2016). *Introduction to random graphs* (Cambridge University Press)

Gao, J., Barzel, B., and Barabási, A.-L. (2016). Universal resilience patterns in complex networks. *Nature* 530, 307–312

Jayasumana, S., Hartley, R., Salzmann, M., Li, H., and Harandi, M. (2015). Kernel methods on riemannian manifolds with gaussian rbf kernels. *IEEE transactions on pattern analysis and machine intelligence* 37, 2464–2477

Kolouri, S., Park, S. R., and Rohde, G. K. (2015). The radon cumulative distribution transform and its application to image classification. *IEEE transactions on image processing* 25, 920–934

Kolouri, S., Zou, Y., and Rohde, G. K. (2016). Sliced wasserstein kernels for probability distributions. In *Proceedings of the IEEE Conference on Computer Vision and Pattern Recognition*. 5258–5267

Kubota, T., Takahashi, H., and Nakajima, K. (2021). Unifying framework for information processing in stochastically driven dynamical systems. *Physical Review Research* 3, 043135

Legenstein, R. and Maass, W. (2007). Edge of chaos and prediction of computational performance for neural circuit models. *Neural networks* 20, 323–334

Mejias, J. F. and Longtin, A. (2014). Differential effects of excitatory and inhibitory heterogeneity on the gain and asynchronous state of sparse cortical networks. *Frontiers in computational neuroscience* 8, 107

Panda, P. and Srinivasa, N. (2018). Learning to recognize actions from limited training examples using a recurrent spiking neural model. *Frontiers in neuroscience* 12, 126

Tu, C., D'Odorico, P., and Suweis, S. (2021). Dimensionality reduction of complex dynamical systems. *Iscience* 24, 101912

Wong, A., Famouri, M., Pavlova, M., and Surana, S. (2020). Tinyspeech: Attention condensers for deep speech recognition neural networks on edge devices. *arXiv preprint arXiv:2008.04245*
